# Associations of Blood Pressure Parameters with Cognitive Decline and Dementia: A Systematic Review of Reviews

**DOI:** 10.1093/ajh/hpaf213

**Published:** 2025-10-30

**Authors:** Sultana Shajahan, Megan Heffernan, Katie Harris, Cheryl Carcel, Mark Woodward, Craig S Anderson, Ruth Peters

**Affiliations:** The George Institute for Global Health, The University of New South Wales, Sydney, Australia; The George Institute for Global Health, The University of New South Wales, Sydney, Australia; The George Institute for Global Health, The University of New South Wales, Sydney, Australia; The George Institute for Global Health, The University of New South Wales, Sydney, Australia; Prince of Wales Clinical School, The University of New South Wales, Sydney, Australia; The George Institute for Global Health, The University of New South Wales, Sydney, Australia; The George Institute for Global Health, Imperial College London, London, United Kingdom; The George Institute for Global Health, The University of New South Wales, Sydney, Australia; Institute of Science and Technology for Brain-inspired Intelligence, Fudan University, Shanghai, China; Neurology Department, Royal Prince Alfred Hospital, Sydney, Australia; The George Institute for Global Health, The University of New South Wales, Sydney, Australia

**Keywords:** blood pressure, cognitive decline, dementia, hypertension, variability

## Abstract

**Background:**

Evidence syntheses on the associations between BP parameters and cognitive decline and/or dementia have taken different methodological approaches and targeted different BP parameters and outcomes. The aim of this umbrella review was to provide a high-level synthesis of published systematic reviews with meta-analyses on these associations.

**Methods:**

PubMed, Embase, PsycINFO, and Cochrane were searched up to April 2025 for eligible reviews. Risk of bias was assessed using the AMSTAR-2 tool, and overlap of constituent studies between reviews was explored.

**Results:**

Among 31 included reviews, 8 reported positive associations between higher BP and greater incidence of cognitive decline or dementia, 5 drew neutral conclusions, and 1 reported an inverse relationship. Greater mid-life BP was associated with greater risk of all-cause dementia, whereas late-life hypertension might have a mixed or overall neutral association. Three reviews reported associations between higher BP variability and all-cause dementia, and 2 for cognitive decline. Reviews also reported associations between higher pulse wave velocity and orthostatic hypotension and poorer outcomes. No reviews examined PP, mean-arterial pressure, or cumulative BP load. Most reviews were of low quality, with considerable heterogeneity in BP parameter definitions and outcome criteria. Overlap of constituent studies for each BP parameter was low.

**Conclusions:**

In addition to high BP, incorporating variability, pulse wave velocity and orthostatic hypertension into risk assessments of cognitive decline or dementia and adopting standardized definitions for BP parameters and cognitive outcomes may improve comparability across future studies and strengthen clinical guidance.

## Graphical abstract

**Graphical Abstract:**
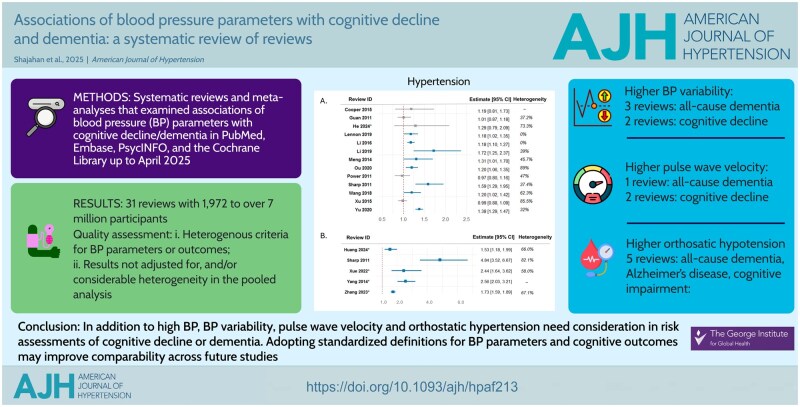

## Introduction

It is estimated that over 55 million people are living with dementia globally, over two-thirds of whom are living in low-and middle-income countries.^1^ This figure is expected to nearly double over the next few decades owing to ongoing ageing of populations.^2^ Dementia is now one of the leading causes of death and disability, affecting approximately 5%-7% of the population, mostly among those aged over 60 years.^2^ Elevated BP is well recognized as being associated with an increased risk of cognitive decline and/or dementia,^3-9^ and there is increasing evidence that BP lowering treatment can reduce these risks.^3-5^^,^^7^^,^^10^

Over the 2 decades since Qiu et al^11^ published their seminal review on the association of BP with cognition/dementia, additional systematic reviews and meta-analysis have sought to draw together epidemiological and/or clinical trial data to refine the precision of effects.^3^^,^^12-14^ However, each evidence synthesis approach has had a slightly different focus, and used different methods and criteria for BP parameters, treatments and outcomes. As the definitions of hypertension have changed as the evidence-base has increased, it would appear appropriate that an overview be undertaken to reevaluate the evidence, assess the risk of bias, synthesize the results, and highlight important new findings in this area.

As the literature around BP and dementia is considerable, it can be difficult for health professionals and policy makers to determine which BP parameters are the most important in defining those at highest risk, and which therapeutic targets are appropriate for preventing cognitive decline and/or dementia. Umbrella reviews or “review of reviews” methodology allows a systematic high-level summary of the evidence. The aim of this umbrella review was to summarize the content and quality of the current body of evidence on the relationship between the most commonly used BP metrics in clinical settings and cognitive decline or dementia, and calculate overlap of constituent studies between reviews.

## Methods

### Registration

A review protocol was developed and registered on the PROSPERO international prospective register of systematic reviews (registration number CRD42023380633), and followed the Preferred Reporting Items for Systematic Reviews and Meta-Analyses guideline.^15^ The protocol provides full details of the methods used and any changes invoked subsequent to original registration.

### Inclusion and exclusion criteria

We included systematic reviews with meta-analyses or meta-regressions which examined associations with prevalent (cross-sectional) or incident (longitudinal) cognitive decline/dementia. Reviews were excluded if they focused on pregnancy-related BP changes (eg, preeclampsia and eclampsia) or distinct subpopulations (eg, those with schizophrenia). The included reviews were restricted to published articles including a systematic search (identified the data source, last date of search, inclusion and exclusion criteria) and analysis plan (how the results were pooled and if any tests for heterogeneity and publication bias were done); gray literature and abstracts from conference presentations were not searched. Analysis plans were scrutinized in the published articles and accompanying supplementary files. Studies using self-reported outcome measures were ineligible, since self-reported dementia-related diagnosis can inaccurately estimate the prevalence of dementia in older adults.^16^

### BP parameters

To capture commonly used and emerging BP parameters that have been reported as having a role in an increased risk of cognitive decline or dementia, the exposures included: hypertension, mean SBP; mean DBP; BP variability; PP; mean arterial pressure (MAP); carotid-femoral pulse wave velocity (PWV); orthostatic hypotension (OH), and cumulative SBP load. Detailed definitions are provided in Appendix S1.

### Outcomes

The primary outcomes of interest were cognitive decline, incidence or prevalence of dementia, or a composite of both. Cognitive function may be measured using any clinical cognitive assessment tool(s), including but not limited to the Mini-Mental State Examination (MMSE), Montreal Cognitive Assessment (MoCA), and neuropsychological tests. Dementia may be diagnosed by any widely accepted diagnostic tools, such as the Diagnostic and Statistical Manual of Mental Disorders, Fourth or Fifth Edition, or International Classification of Diseases (ICD) codes.

### Databases and selection of studies

PubMed, Embase, PsycINFO, and Cochrane databases were searched on April 15, 2025, using a combination of key words and MeSH terms for BP, hypertension, PWV, variability, MAP, PP, cognitive dysfunction, dementia, and Alzheimer’s disease (AD) (details are provided in Appendix S2). Two reviewers (S.S. and M.H.) independently selected studies against the eligibility criteria and any conflicts between the 2 reviewers were resolved by a senior third reviewer (R.P.).

### Data extraction, quality assessment, and synthesis

Data were extracted in duplicate (S.S. and M.H.) and comprised the following items: lead author and publication year, methodology (systematic reviews with meta-analyses and/or meta-regression), number and sample size of constituent studies, range of follow-up lengths and population age and sex breakdown in each review/meta-analysis, dates/databases and search terms used to populate reviews or source of data/key data parameters for meta-analyses, BP measures, measures of diagnosis of cognition and dementia, statistical measures, assessment of bias, pooled relative risk (RR), or similar, for cognitive impairment or dementia (unadjusted and adjusted) with 95% confidence intervals (CIs), and list of covariates if adjusted results were included. Reviews were evaluated in duplicate using the AMSTAR 2 checklist (Appendix S3). Any disagreements were resolved by discussion with a senior author (R.P.). The data extracted and findings were summarized in a narrative synthesis including detailed tables. Overlap of the constituent studies across reviews reporting the same BP parameters was quantified using the Corrected Covered Area Index.^17^

## Results

### Search results

**Figure 1.**
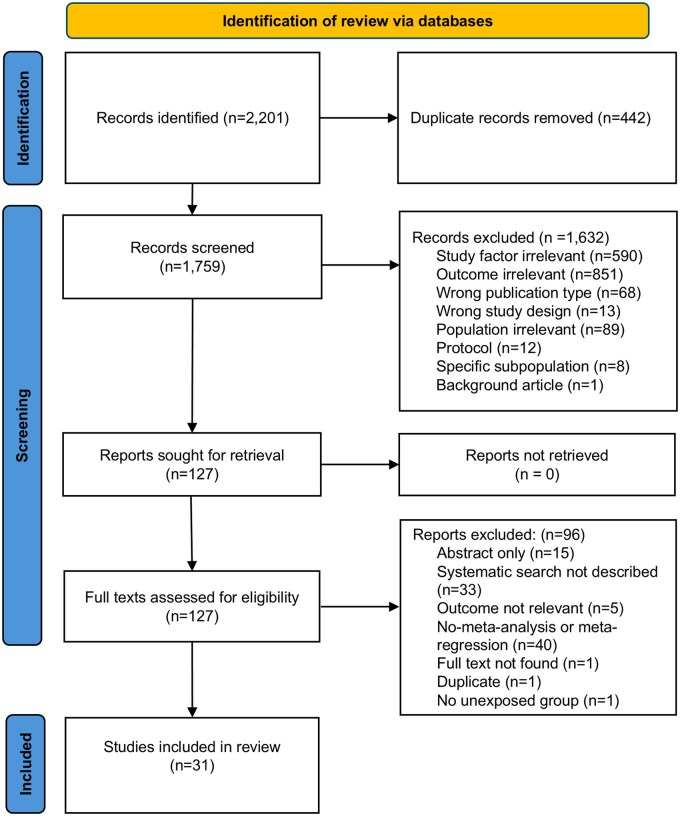
Flow chart showing the study selection process.

In total, 2,201 articles were identified from database searching (Figure 1). After removing 442 duplicates, 1,759 articles remained. After title abstract screening, 127 articles were identified for full text screening. Finally, 31 reviews were eligible for inclusion. A list of excluded studies after full text screening is provided in supplementary Table S1.

### Characteristics of the included reviews

Table 1 summarizes the characteristics of the included reviews that covered various BP parameters for hypertension,^3^^,^^14^^,^^18-30^ SBP and/or DBP,^14^^,^^22^^,^^27^^,^^31-36^ BP variability,^14^^,^^37^^,^^38^ PWV,^39-41^ and OH.^42-44^ No reviews were found for PP, MAP, or cumulative BP load. Reviews included constituent studies ranging in size from 3^19^ to 323,^36^ with the total numbers of participants ranging from 1,972 to over 7 million. Two reviews focused on only participants with mild cognitive impairment (MCI),^3^^,^^20^ 1 included either those with normal cognition or MCI,^14^ 1 included participants with hypertension only,^31^ and 2 included only older participants, age ≥ 65^42^ or 50-80^23^ years. Eighteen of the 31 reviews were published in the last 5 years.^14^^,^^19^^,^^21^^,^^23^^,^^25-30^^,^^32^^,^^33^^,^^37-4^,^4^,^43^^,^^45^ Nineteen reviews examined dementia as an outcome,^3^^,^^14^^,^^18-22^^,^^24^^,^^25^^,^^32-38^^,^^40^^,^^43^^,^^44^ while the others examined cognitive ecline overall or decline within specific cognitive domains.^14^^,^^23^^,^^26-31^^,^^37-43^^,^^45^ Twenty-two reviews excluded baseline impairment and/or dementia, or explicitly stated that they investigated incident dementia.^3^^,^^14^^,^^18^^,^^20-28^^,^^31^^,^^33-37^^,^^41^^,^^43-45^ Only 1 review combined cognitive decline or dementia as an outcome.^45^ Two reviews included trials combined with observational studies,^25^^,^^45^ 19 reviews included only cohort studies with longitudinal follow-up and/or nested case-control studies,^3^^,^^14^^,^^18-22^^,^^26^^,^^28^^,^^32-38^^,^^41^^,^^43^^,^^44^ and 10 reviews included both cross-sectional and longitudinal articles.^23^^,^^24^^,^^27^^,^^29-31^^,^^39^^,^^40^^,^^42^^,^^45^ No reviews were restricted to a single sex. Most reviews drew constituent studies from North American, European and Chinese populations, but some included Australian, Taiwanese, Japanese and Korean populations;^14^^,^^19^^,^^22^^,^^33^^,^^35^^,^^36^^,^^38^ 2 studies did not specify a particular geographical distribution.^23^^,^^31^ Further details are provided in supplementary Table S2.

**Table 1.** Characteristics of the included systematic reviews and meta-analyses.

Review ID	Country of study	Population stated as included in the review	Actual mean age range of constituent studies (years)	Source of evidence reported by the reviews	BP parameters identified	Definition of BP parameters	Outcome studied	Criteria for outcome assessment	Follow-up (years)	Number of studies
***Reviews including longitudinal data***										
**Chiu et al (2021)^37^**	Taiwan	General population without dementia at baseline	54.3-84.4	Longitudinal studies	BPV	Ambulatory, home, or visit-to-visit BP monitoring using CV; SD; VIM; average real variability (ARV)	Cognitive decline and dementia	Cognitive decline: MoCA, CAMCOG Dementia: DSM-IV, DSM-III-R, NINCDS-ADRDA, NINDS-AIREN, or ICD-10	1-26	8
**Cooper et al (2015)^3^**	United Kingdom	People with MCI, in the absence of dementia	Not specified	Longitudinal studies	Hypertension	Not specified	Incident dementia	All cause dementia, Alzheimer’s dementia, or other dementias separately	1.5-9	7
**Guan et al (2011)^18^**	China	General population	40- >75	Longitudinal studies	Hypertension	SBP ≥160 mm Hg or DBP ≥90 mm Hg	Alzheimer’s disease (AD)	Not specified	1-32	9
**He et al (2024)^26^**	China	Community-dwelling adults aged 55 years or older with normal cognitive function at baseline	Not specified	Longitudinal studies	Hypertension	Not specified	MCI	Revised Mayo Clinic Criteria for MCI, Peterson criteria, Winblad criteria, Neuropsychological test battery, Recommendations of the National Institute on Ageing- Alzheimer’s Association^a^	Not specified	3
**Jia et al (2021)^38^**	Hong Kong	Any adults	50.9-79.9	Longitudinal studies	BPV	BPV using: SD, CV, VIM, and ARV	Dementia or cognitive decline	DSM III or IV, NINCDS-ADRDA, MMSE, MoCA^b^	14	16
**Joyce et al (2024)^28^**	Ireland	Adults aged 40-65 years	48.6-58.6	Longitudinal studies	Mid-life hypertension	Hypertension diagnosis by clinician, self-report, and/or by recorded BP metric in line with accepted definitions: the ESC definition (*n* = 30), American Heart Association (AHA) (*n* = 8), use of antihypertensive medication (*n* = 12), and self-reported hypertension (*n* = 10); 7 studies did not specify a working definition	Cognitive decline in memory, attention, executive function, and global cognition	Tests for each domain^c^	Not specified	15
**Lee et al (2022)^32^**	United Kingdom	Not specified	Not specified	Longitudinal studies	High BP	Not specified	Dementia	Not specified		12
**Lennon et al (2019)^19^**	Australia	Any population	50.4-63	Longitudinal studies (prospective cohort or nested case control)	Hypertension	Systolic hypertension: Stage 1 > 140 mm Hg, Stage 2 > 160 mm Hg, Diastolic hypertension: >90 mm Hg, Increments in BP by 10 mm Hg	AD	DSMIV, ICD-9, NINDS	13-22	3
**Li et al (2016)^20^**	United States	With MCI at baseline	70-80.7	Longitudinal studies	Hypertension	Not specified	Both AD and dementia	Not specified	Not specified	60
**Li et al (2019)^33^**	China	Adults aged 35-65 years	49.2-57	Longitudinal studies	SBP	High SBP ≥ 160 mm Hg, Borderline 140 ≤ SBP-160 mm Hg	Dementia	Not specified	12.8-40	7
**Liang et al (2020)^21^**	China	Aged >50 years with no dementia at baseline	73.7-76.4	Longitudinal population-based studies (prospective/retrospective)	Hypertension	Not specified	Incident dementia	Not specified		9
**Meng et al (2014)^22^**	China	Aged 40-65 years	43-60	Longitudinal studies	Hypertension	SBP/DBP ≥160/95 mm	AD	NINCDS-ADRDA criteria	13.6-37	5
**Min et al (2020)^43^**	China	Adults with no dementia at baseline	45-83.5	Prospective longitudinal studies	OH	1996 consensus statement of OH	Dementia, cognitive impairment (CI), cognitive decline (CD)	Dementia: ICD-9, DSM-III-R, DSM-IV CI: defined as MMSE <23 in 1 study, as a score of 0 or 1 on the 3 words delayed recall task of the MMSE in another, and as MMSE ≤24 CD: defined as a drop in MMSE score of ≥1 point in 1 study, as a MMSE score drop of ≥3 points in another study	2-28 years	Total = 13 for worse cognition: - 6 for dementia, 5 for CI, 2 for CD
**Ou et al (2020)^14^**	China	Participants with normal cognition or MCI	35.3-93.2	Prospective longitudinal studies (cohort, case-cohort, or nested case-control)	Hypertension, SBP, DBP, prehypertension, BPV, BP change, OH, and PP	Hypertension: SBP ≥130 mm Hg, DBP ≥80 mm Hg, or use of antihypertensive medications Prehypertension: SBP between 120-139 mm Hg, DBP between 80 and 89 mm Hg^d^	Dementia, cognitive impairment	Not specified	1.5-43	136
**Pase et al (2012)^41^**	Australia	Any population	54-87	Longitudinal studies	Arterial Stiffness	Validated measure of large artery stiffness, central pressure, wave reflections or large artery compliance	Cognitive decline	Valid assessment of cognitive decline or dementia	5	4
**Peters et al (2018)^44^**	Australia	Any population	45-73.4	Prospective longitudinal studies	OH	Multiple definitions, most common definition was a fall of ≥20 mm Hg SBP and/or a fall of ≥10 mm Hg DBP after supine rest for 10 minutes and standing for 1 minute	Dementia (used standard diagnostic criteria)	MMSE, neuropsychological battery tests, DSM-IV, DSM-III-R	1-28	5
**Power et al (2011)^34^**	United States	Any population	50-82	Prospective longitudinal studies (cohort studies or nested case-control studies)	SBP, DBP	SBP >130 mm Hg DBP >85 mm Hg or self-reported antihypertensive medication use	AD	Not specified	2-27	
**Wang et al (2018)^35^**	China	Any population	49-94	Prospective longitudinal studies (nested case-control or prospective cohort studies)	SBP, DBP, hypertension, PP	Not specified	Dementia	No diagnostic criteria provided	2.1-32	21
**Xu et al (2015)^36^**	China	General population	49.6-79.8	Longitudinal cohort or retrospective case-control studies	High SBP, Low DBP	SBP ≥160 mm Hg, Low DBP (at least <70 mm Hg)	AD	Not specified	Not specified	323
**Yu et al (2020)^25^**	China	No restriction on population except for MCI and special populations (special populations not specified)	Not specified	Prospective longitudinal studies	Mid-life hypertension, OH	Not specified	AD	AD independently diagnosed according to the NINCDS-ADRDA criteria, or an RCT—incidence of AD or AD-related clinical endpoints (dementia or cognitive impairment)	Not specified	Not specified
***Reviews including cross-sectional and longitudinal data***										
**Alvarez-Bueno et al (2020)^39^**	Spain	Adults aged 46-85 years	46-85	Cross-sectional and longitudinal studies	PWV	Measured using carotid-femoral, brachial-ankle or aortic PWV	Cognitive domains (global cognition, executive function and memory)	Using MMSE and other neuropsychological tests^e^	Not specified	38
**de Heus et al (2021)^45^**	The Netherlands	Adults aged ≥ 18 years	66-80	Case-control, prospective cohorts, database registries, cross-sectional, and secondary analyses of RCTs	BPV	BPV using repeated measurements of BP at rest	Dementia or cognitive decline	Dementia was criterion-referenced ICD criteria, DSM criteria, an adjudicated expert panel or the prescription of antidementia drugs, and cognitive impairment defined using standardized criterion	1-13	20
**Gifford et al (2013)^31^**	United States	Participants with hypertension	43-91	Observational (cross-sectional or longitudinal prospective study but not RCTs or intervention studies)	SBP, DBP	Elevated SBP >140 mm Hg and/or DBP >90 mm Hg	Cognitive decline	MMSE and other neuropsychological tests^f^	Not specified	8
**Huang et al (2024)^27^**	China	Adults aged ≥18 years with cognitive impairment after stroke (ischemic or hemorrhagic)	59.9-75.17	Longitudinal and cross-sectional combined for hypertension, Only longitudinal for SBP and DBP	Hypertension, SBP	Definition of hypertension not specified. SBP levels <120 mm Hg, 120-139 mm Hg, 140-159 mm Hg, 160-179 mm Hg, ≥180 mm Hg	Cognitive impairment	Clinical Dementia Rating (CDR), Clinical diagnosis at the final follow-up visit, Hodkinson Abbreviated Mental Test (HAMT), MMSE, Clock Drawing Test, MoCA, Vascular Neuropsychological Battery (V-NB)	Discharge-4.4 years	For hypertension = 6 studies For SBP = 12 studies
**Iseli et al (2019)^42^**	Australia	Adults aged ≥ 65 years	60.9-88.3	Cross-sectional and longitudinal studies	OH	Decrease of at least 20 mm Hg SBP and/or 10 mm Hg DBP within the first 3 minutes of standing	Cognition	MMSE and other neuropsychological tests^g^	Not specified	16
**Liu et al (2021)^40^**	China	Adults aged >18 years	46-80	Cross-sectional or longitudinal studies	Aortic PWV	Validated PWV measurement along aorta	Cognitive decline and dementia	Validated scales and clinic diagnostic standards or guidelines	1-15	29
**Sánchez-Nieto et al (2021)^23^**	Mexico	Elderly aged 50-80 years	34.9-79	Cross-sectional and longitudinal studies (cohort or case-control)	Arterial hypertension	Medical diagnosis or by antihypertensive medication use	Cognitive decline	At least 2 valid neuropsychological instruments to measure cognitive functions. Cognitive domains were assessed using different tests across studies^h^	Not reported	8
**Sharp et al (2011)^24^**	United Kingdom	Total dementia population	57.8-80.4	Cross-sectional and longitudinal studies	Arterial hypertension	Hypertension based on either a prior diagnosis or a cross-sectional BP measurement	Vascular dementia	DSM III or IV, ICD-10, Information-memory-concentration, NINDS-AIREN, Hachinski Ischemia score, Rosen Ischemia score	3.2-10	6
**Xue et al (2022)^29^**	China	Patients with type 2 diabetes	Not specified	Cross-sectional and longitudinal studies	Hypertension	Not specified	MCI	MoCA, MMSE, European Alzheimer’s Disease Consortium 2006 MCI criteria, Petersen’s criteria, Chinese expert consensus on cognitive disorder diagnosis and treatment	Not specified	7
**Yang et al (2014)^46^**	China	Chinese population with vascular cognitive impairment (VCI)	Not specified	Cross-sectional and longitudinal studies	Hypertension	Not specified	VCI	Not specified	Not specified	42
**Zhang et al (2023)^30^**	China	Chinese elderly aged ≥60 years	Not specified	Case-control or cohort studies	Hypertension	Not specified	MCI	MoCA, MMSE, Petersen’s criteria, Chinese expert consensus, 2006 European Alzheimer’s Disease Consortium MCI criteria	Not specified	Not specified

Abbreviations: BPV, BP variability; VIM, variance independent of the mean; ARV, average real variability; MoCA, Montreal Cognitive Assessment; CAMCOG, Cambridge Cognition Examination; DSM-IV, The Diagnostic and Statistical Manual of Mental Disorders, Fourth Edition; DSM-III-R, The Diagnostic and Statistical Manual of Mental Disorders, Third Edition; NINCDS-ADRDA, National Institute of Neurological and Communicative Disorders and Stroke and the Alzheimer’s Disease and Related Disorders Association; NINDS-AIREN, National Institute of Neurological Disorders and Stroke-Association Internationale pour la Recherche et l‘Enseignement en Neurosciences; ICD-10, International Code of Diseases Tenth Edition; MCI, mild cognitive impairment; PWV, pulse wave velocity; AD, Alzheimer’s disease; MMSE, Mini-Mental State Examination; OH, orthostatic hypotension.

a Revised Mayo Clinic Criteria for MCI 1. Cognition concern by the participant, informant, coordinator, or physician; 2. Impairment in 1 or more neuropsychological domain; 3. Essentially normal functional activities as derived from the Clinical Dementia Rating scale and the Functional Activities Questionnaire; and 4. Absence of dementia. Peterson criteria 1. Memory problems, 2. Objective memory disorder, 3. Absence of other cognitive disorders or repercussions on daily life, 4. Normal general cognitive function and 5. Absence of dementia. Winblad criteria (2009) determined MCI as performance on a single cognitive test within a domain >1.5 SD below normative expectations in the Neuropsychological test battery. Participants were classified as MCI subtypes: 1. Single Domain Amnestic if only the memory domain was impaired, 2. Single Domain Non-Amnestic if the memory domain was not impaired and only 1 nonmemory domain was impaired, 3. Multiple Domain Amnestic MCI if memory and at least one other domain showed impairment, and 4. Multiple Domain Non-Amnestic if the memory domain was not impaired but more than 1 nonmemory domain was impaired. Recommendations of the National Institute on Ageing- Alzheimer’s Association (Lara et al., 2017). 1. Concern about a change in cognition 2. Objective evidence of impairment in 1 or more cognitive domains based on a 1-SD cutoff after adjusting for level of education and age of the sample. 3. Preservation of independence in functional abilities; and 4. No dementia.

b Diagnosed according to Diagnostic and Statistical Manual of Mental Disorders (III or IV), or the criteria of the National Institute of Neurological and Communication Disorders and Stroke and of the Alzheimer Disease and Related Disorders Association, or antidementia drugs prescribed at least 2× and the codes with Alzheimer disease (International Classification of Disease, Tenth Revision [ICD-10] F00, or G30), vascular dementia (ICD-10 F01) as well as other dementia (ICD-10 F02, F03, G23.1, or G31). The incidences of cognitive impairment were measured by a cutoff of MMSE score ≤24, MoCA <26 or a decline >1.5 standard variation. The cognitive decline was defined as the MMSE or MoCA score change during the follow-up period.

c Memory: Delayed Recall (various forms: Verbal Memory, Logical Memory, RAVLT), Immediate Recall, East Boston Memory Test (EBMT), California Verbal Learning Test (CVLT), CERAD Word List Recall/Recognition, Digit Span Backward (DSBT/DSB), Logical Memory Subtest, Visual Reproduction—Delayed (VR-d Test), McNair Survey Memory Scale. Working Memory/Attention/Processing Speed: Digit Symbol Substitution Test (DSST/SDMT), WAIS Digit Sequencing or Digit Span, Trail Making Test A & B (TMT-A/TMT-B), Stroop Test, Symbol Digit Modalities Test (SDMT), 5-Choice Movement Time Task, CMS Working Memory Index, Continuous Recognition Paradigm. Executive Function/Verbal Fluency: Verbal Fluency Test (VFT/Category Fluency), Controlled Oral Word Association Test (COWAT), Modified Boston Naming Test (BNT), Stroop Interference Test, AH-4 Inductive Reasoning Test. Global Cognition: Mini-Mental State Examination (MMSE), Montreal Cognitive Assessment (MoCA), Informant Questionnaire on Cognitive Decline in the Elderly (IQCODE), HRS Cognitive Score, CAMCOG (Cambridge Cognitive Examination), CAIDE Risk Score (used as a proxy for cognitive aging in some studies). Visuospatial & Intelligence Tests: Hooper Visual Organization Test, Constructional Praxis Test (and Recall), WAIS—General Intelligence subscales, National Adult Reading Test (NART).

d OH SBP drop of at least 20 mm Hg or a DBP drop of at least 10 mm Hg after 3 minutes postural change. PP: The difference between SBP and DBP. BPV: Coefficient of variation (SD/mean BP) for both SBP and DBP. DBP change: DBP level at re-examination minus the level at baseline.

e Tests used to measure cognitive function aimed to measure global cognition, executive function, memory, language, attention, processing speed, and visuospatial ability: MMSE, Trail Making Tests (A and B), Verbal Fluency Test, Wechsler Adult Intelligence Scale, Revised Digit Span, 3MS, Montreal Cognitive Assessment, California Verbal Learning Test, Digit Symbol Substitution Test, Figure Comparison, and Stroop Test (parts I and II), Digits Backward and the Stroop Test (partIII), Block Design, Object Assembly, Visual Reproductions Immediate and Delayed, Hooper Visual Organization Test, Matrix Reasoning, Symbol Search, Logical Memory Immediate and Delayed, Hopkins Verbal Learning Test, Digit Span Forward and Backward, Letter-Number Sequence, Controlled Oral Word Associations, Stroop Color Word Test (parts I and II), the Concept Shifting Test Part A and B, and the Letter-Digit Substitution Test, Stroop Color Word Test (partIII) and the Concept Shifting Test Part C, Letter Digit Substitution test, Mental flexibility, SPOTING the symbol, Digit Span Forward, Executive Function Test, Focused Attention, Sustained Attention, Delayed Memory Recall, Visual Spatial Memory, Visual Spatial Short Term Recall, Cognitive Efficiency Profile, K-MMSE, Color-Word Interference Stroop Task, Letter Digit Substitution Test, Purdue Pegboard Test, Rey Auditory Verbal Learning Test, Story Memory and Recall, Boston Naming Test, Brief Visuospatial Memory Test Revised, Digit Span-Backward, Block Design, Color Trails Test 2, Categorical Verbal Fluency, California Verbal Learning Test, Digits Forward, Digit Symbol Substitution Test, Figure Comparison, Cambridge Neuropsychological, Test Automated Battery Spatial Working Memory Rey-Osterrieth Complex Delayed Recall, Semantic Verbal Fluency animal category Phonological Verbal Fluency, Clock Drawing Test, Doors Test, List of nouns Dutch Adult Reading Test, Quick test of cognitive speed (AQT), Logical memory delayed, Incidental learning, Victoria Stroop interference task, Visual Reproductions delayed Hooper visual organization test (VOT), Letter-Digit Substitution Task, Word Fluency Test, Seoul Neuropsychological Screening Battery, Rey Complex Figure Test, Calculation test, Seoul Verbal Learning Test, Control Oral Word, Association Test, Benton Visual Retention Test, Animal Naming test, Phonemic Fluency (FAS), Block Design, Similarities test.

f Global cognition score was measured using a composite score or MMSE; episodic memory was assessed via the California Verbal Learning Test-II, Consortium to Establish a Registry for Alzheimer’s Disease (CERAD) Word List Immediate and Delay Recall, or the Wechsler Memory Scale (WMS). Language was assessed via the Animal Fluency, Boston Naming Test, or Category Fluency, Attention via the Wechsler Adult Intelligence Scale (WAIS), Executive Functioning via Clock Drawing, Composite Score, or WAIS or WMS-R digit span, etc, information processing speed by the Trails (Motor speed and scanning subtests), Trails A, WAIS-R Digit Symbol Coding, or the WAIS-III Digit Symbol Coding, visuoperceptual skills was assessed using the Composite Score, WAIS-R Block Design, Figure-copying Test, Judgment of Line Orientation, or Pattern Comparison Test.

g Clinical Dementia Rating (CDR), Cognitive State Test (COST), Wechsler Test of Adult Reading (WTAR), Stroop test, Digit Span Test, Arithmetic test, Verbal fluency, Symbol search test, Consortium to Establish a Registry for Alzheimer’s Disease (CERAD) verbal learning test, Verbal fluency, 10-word recall task, Visual reasoning tasks, Color Trail, Choice reaction time (CRT), Word recall test, Picture memory test, Sustained Attention to Response Task (SART), Repeatable Battery for Assessment of Neurological Status (RBANS), Cognitive and self-contained part of the Cambridge Examination for Mental Disorders of the Elderly (CAMCOG), Frontal Assessment Battery (FAB), Seoul Neuropsychological Screening Battery, Hasegawa Dementia Scale Revised (HDSR), Visuospatial Cognitive Performance Score (VCPS), Cognitive Efficiency Profile (CEP), Neuropsychological battery, Informant questionnaire on cognitive decline in the elderly (IQCODE), Hachinski ischemia scale, Geriatric Mental State Schedule (GMS), Cambridge Examination for Mental Disorders of the Elderly (CAMDEX).

h Verbal fluence; phonological verbal fluency; semantic verbal fluency; Digit Span Backwards test; letter cancellation test, Wisconsin card test; Symbol Digit Modalities Test; MMSE, East Boston Memory Test; Temporal orientation; Image copy; Trail Making Test B; Trail Making Test A; Stroop color-word test, Letter Number Sequence test; Digit Span Forward; numeric work memory; arithmetic; Alpha Span; visual memory span backwards; Digit-symbol substitution; manual speed motor; Reaction time in retention and memory tests; Symbol Digit Modalities Test; Letter comparison; Wordlist (number of words); Delayed wordlist (waiting time); History memory; Delayed history memory; Visual memory; Delayed visual memory; Cognitive Drug Research Computerized Assessment; Rey complex figure test-immediate; Rey Complex Figure Test-delayed; Raven Matrices; Mathematical Reasoning; Blok Desing; Picture Completion; Picture arrangement; Object Assembly; Clock Drawing Test; and Every problem solving test.

### Associations of BP parameters with cognitive outcomes

#### Hypertension

Twenty-one systematic reviews reported results for hypertension or raised BP. Fourteen reviews included meta-analyses of only longitudinal data to report on incident dementia (all-cause), AD, or vascular dementia (VD),^3^^,^^14^^,^^18-22^^,^^24^^,^^25^^,^^32-36^ 1 reported longitudinal and cross-sectional associations separately for VD,^24^ 3 reported longitudinal associations with cognitive decline,^14^^,^^26^^,^^28^ and 6 combined cross-sectional and longitudinal studies in their meta-analyses and reported on the prevalence of cognitive decline^23^^,^^27^^,^^29-31^^,^^46^ (Tables 2 and 3).

**Table 2.** Meta-analysis results from constituent reviews showing associations of BP parameters with dementia.

Review ID	Type of meta-analysis	BP parameters identified	Dementia type	Univariate	Multivariate (if applicable)	Adjustments (if applicable)	Overall result
***Reviews including longitudinal data***							
**Chiu et al (2021)^37^**	Published data	BPV, office or home BP	All-cause dementia, AD and vascular dementia		**SBP: All-cause dementia** CV = hazard ratio (HR) 1.45 (95% CI, 1.11-1.90), *I*^2^ = 78% SD = 1.31 (95% CI, 1.03-1.67), *I*^2^ = 70% VIM = 1.44 (95% CI, 0.87-2.40), *I*^2^ = 82% **DBP: All-cause dementia** CV = 1.64 (95% CI, 0.96-2.81), *I*^2^ = 87% **SBP: Alzheimer’s dementia** CV = 1.51 (95% CI, 0.80-2.86), *I*^2^ = 83% SD = 1.47 (95% CI, 0.81-2.68), *I*^2^ = 77% VIM = 1.46 (95% CI, 0.82-2.57), *I*^2^ = 80% **DBP: Alzheimer’s dementia** CV = 1.71 (95% CI, 0.68-4.28) *I*^2^ = 90% **SBP: Vascular dementia** CV = 1.57 (95% CI, 0.71-3.46), *I*^2^ = 66% SD = 1.83 (95% CI, 0.59-5.63), *I*^2^ = 71% VIM = 1.24 (95% CI, 0.96-1.60), *I*^2^ = 16% **DBP: Vascular dementia** CV = 1.78 (95% CI, 0.62-5.11), *I*^2^ = 75%	Differed by study, including sex, study center, education, diabetes, history of vascular diseases, antihypertensive drug at baseline, and mean BP	**Higher systolic BPV (measured in CV and SD) was associated with greater risk of all-cause dementia**
**Cooper et al (2015)^3^**	Published data	Hypertension	Only all-cause was pooled	OR 1.19 (95% CI, 0.81-1.73)			No relationship between hypertension and all-cause dementia
**Guan et al (2011)^18^**	Published data	Hypertension, SBP ≥160 mm Hg or DBP ≥ 90 mm Hg	AD	RR 1.01 (95% CI, 0.87-1.18), *I* ^2^ = 37.2%			No relationship between hypertension and AD
**Jia et al (2021)^38^**	Published data	BPV	All-cause dementia	**Systolic BPV:** **Visit-to-visit HR** 1.11 (95% CI, 1.05-1.17), *I*^2^ = 53% **Day-to-day** 1.38 (95% CI, 1.23-1.55) **Diastolic BPV:** **Visit-to-visit** 1.14 (95% CI, 1.04-1.25) **Day–to-day** 1.38 (95% CI, 1.23-1.55)			**Both SBP and DBP variability associated with greater risk of all-cause dementia**
**Lee et al (2022)^32^**	Published data	SBP	Both all-cause dementia and late-onset AD pooled	SBP: OR 0.91 (95% CI, 0.64-0.99), *I* ^2^ = 92.6%			**Higher SBP associated with lower risk of all cause dementia**
**Lennon et al (2019)^19^**	Published data	Hypertension	AD		Systolic hypertension >160 mm Hg: HR 1.25 (95% CI, 1.06-1.47), *P* = 0.0065; Systolic hypertension > 140 mm Hg: 1.18 (95% CI, 1.02-1.35), *P* = 0.021	Differed between studies, including age, sex, education, cholesterol, non-fasting blood glucose, glomerular filtration rate, body mass index (BMI), waist-to-hip ratio, pulse	**Hypertension associated with greater risk of AD**
**Li et al (2016)^20^**	Published data	Hypertension	AD	RR 1.18, (95% CI, 1.1-1.27), *P* = 0.66, *I* ^2^ = 0%			**Hypertension associated with greater risk of AD**
**Li et al (2019)^33^**	Published data	High SBP	All-cause dementia	High SBP: RR 1.72 (95% CI, 1.25-2.37), *P* = 0.16, *I*^2^ = 39% Borderline BP: 1.41; (95% CI, 1.23-1.62); *P* = 0.35, *I*^2^ = 10%			**Higher SBP associated with greater risk of all-cause dementia**
**Liang et al (2020)^21^**	Published data	Hypertension	All-cause dementia	OR 0.80 (95% CI, 0.65-0.96)^a^			**Hypertension had a significantly higher risk of all-cause dementia**
**Meng et al (2014)^22^**	Published data	Hypertension, mid-life SBP or DBP	AD	**Hypertension (high SBP or high DBP):** Combined OR 1.1 (95% CI, 0.88-1.37), *P* = 0.41 **High SBP:** 1.77 (95% CI, 0.93-3.37), *P* = 0.08 **High DBP:** 2.38 (95% CI, 1.34-4.23), *P* = 0.00) **Combined OR for high BP**: 1.31 (95% CI, 1.01-1.70), *I* ^2^ = 45.7%			**Combined high SBP or DBP was associated with increased risk of Alzheimer’s dementia** No relationship between high SBP or hypertension and Alzheimer’s dementia **High DBP associated with increased risk of AD**
**Min et al (2020)^43^**	Published data	OH	All-cause dementia		1.30 (95% CI, 1.143-1.48), *I* ^2^ = 31%	Not specified	**OH associated with a greater risk of all-cause dementia**
**Ou et al (2020)^14^**	Published data	Hypertension, SBP, DBP, BPV, BP change, OH	All-cause dementia	**Mid-life hypertension:** RR 1.20 (95% CI, 1.06-1.35), *I*^2^ = 89% **High SBP** 1.54 (95% CI, 1.25-1.89), *I*^2^ = 0% **High DBP** 1.50 (95% CI, 1.04-2.16), *I*^2^ = 47%) **Excessive DBP change** 1.65 (95% CI, 1.28-2.11), *I*^2^ = 0% **Late-life hypertension** 1.02 (95% CI, 0.94-1.10), *I*^2^ = 32% **High SBP in late life** 1.01 (95% CI, 0.81-1.40), *I*^2^ = 65% **High DBP in late life** 0.77 (95% CI, 0.59-1.00), *I*^2^ = 47% **Excessive diastolic BPV in late life** 2.09 (95% CI, 1.27-3.44), *I*^2^ = 57% **Excessive systolic BPV** RR: 1.99 (95% CI, 1.46-2.29), *I*^2^ = 0% **OH:** 1.26 (95% CI, 1.09-1.45), *I*^2^ = 32%			**Mid-life hypertension associated with greater risk of all-cause dementia** **Late life hypertension: no relationship with all-cause dementia** **Greater BPV associated with greater risk of all-cause dementia** **OH associated with a greater risk of all-cause dementia**
**Peters et al (2018)^44^**	Individual patient data	OH	All-cause dementia		RR 1.21 (95% CI, 1.09-1.35), *I* ^2^ = 10.4%	Differed between studies, various covariates including but not limited to age, race/center, gender, education, SBP, DBP, antihypertensive medication, diabetes, ratio of total cholesterol to high density lipoprotein (HDL) cholesterol, lipid lowering medication, smoking status, alcohol intake, anticholinergic medication, BMI, and APOE genotype.	**OH associated with greater risk of all cause dementia**
**Power et al (2011)^34^**	Published data	SBP, DBP	AD	History of hypertension: RR 0.98 (95% CI, 0.80-1.19), *I*^2^ = 42% Combined history of hypertension and hypertension at enrolment: 0.97 (95% CI, 0.80-1.16), *I*^2^ = 47% A 10 mm Hg-increase in SBP: 0.95 (0.91-1.00), *I*^2^ = 0% A 10 mm Hg-increase in DBP 0.94 (0.85-1.04), *I*^2^ = 14%			No relationship between a history of hypertension, current hypertension, higher SBP or DBP and AD
**Wang et al (2018)^35^**	Published data	Hypertension, SBP, DBP	All-cause AD Vascular dementia		All-cause pooled results RR: 1.20 (95% CI, 1.02-1.42), *I*² = 62.3%, *P* = 0.001 Mid-life <65 years: Hypertension and AD: 1.18 (0.75-1.85), *P* = NA, *I*^2^ = NA Hypertension and vascular dementia: 7.68 (3.50-16.84), *P* = NA, *I*^2^ = NA Late life ≥65 years: No significant association with AD, only VD: 3.69 (1.57-8.72), *P* = 0.25, *I*^2^ = 24.7% Late life 75-85 years: High DBP and AD: 0.52 (0.32-0.85), *P* = 0.27, *I*^2^ = 18.7 Very late life >85 years: Hypertension and all-cause dementia: 0.67 (0.48-0.94), *P* = NA, *I*^2^ = NA	Differed between studies, including age, gender, antihypertensive treatment, smoking, diabetes, prevalent cardiovascular disease, and plasma cholesterol	**Greater SBP associated with greater risk of all cause dementia** **Late life hypertension or high DBP may have a protective effect on dementia risk**
**Xu et al (2015)^36^**	Published data	SBP ≥160 mm Hg, Low DBP (at least <70 mm Hg)	AD	Combined OR and RR: SBP ≥160 mm Hg: 0.99 (0.88-1.09), *I*^2^ = 85.5% Low DBP 1.18 (0.97-1.39), *I*^2^ = 4.6%			**Hypertension associated with a greater risk of AD**
**Yu et al (2020)^25^**	Published data	Hypertension in mid-life, OH	AD		Hypertension in mid-life: RR 1.38 (95% CI, 1.29-1.47) OH: 1.18 (1.02-1.35), *I*^2^ = 0%	Not specified	**Mid-life hypertension associated with a greater risk of AD** **OH associated with a greater risk of AD**
***Reviews including cross-sectional and longitudinal data***							
**Liu et al (2021)^40^**		PWV	All-cause dementia		Highest category of aortic PWV: 2.10 (95% CI, 1.16-3.80), *I* ^2^ = 64.7, *P* = 0.06		**PWV associated with all-cause dementia**
**Sharp et al (2011)^24^**	Published data	Arterial hypertension	Incidence of vascular dementia	OR 1.59 (95% CI, 1.29-1.95), *I* ^2^ = 37.4			**Hypertension associated with incidence of vascular dementia**
			Prevalence of vascular dementia	OR: 4.84 (95% CI, 3.52-6.67), *P* < 0.01			**Hypertension associated with prevalence of vascular dementia**

Note: The significant findings from each study are highlighted in bold.

Abbreviations: BPV, BP variability; AD, Alzheimer’s disease; CV, coefficient of variation; HR, hazard ratio; CI, confidence interval; SD, standard deviation; VIM, variation independent of the mean; OR, odds ratio; RR, relative risk; OH, orthostatic hypotension; PWV, pulse wave velocity.

a Bayesian network meta-analysis, using the observation group as the reference, patients with hypertension experienced a significantly increased risk of all-cause dementia compared with the observation group. The interpretation depends on the Bayesian framework and the reference group—those without hypertension had a lower risk (0.80), reinforcing the idea that hypertension is a risk factor for dementia.

**Table 3.** Meta-analysis results from constituent reviews showing associations of BP parameters with cognitive decline.

Review ID	Type of meta-analysis	BP parameters identified	Unit of cognition	Univariate	Multivariate (if applicable)	Adjustments (if applicable)	Overall result
**Chiu et al (2021)^37^**	Published longitudinal cohort studies	BPV, office or home BP	Incidence of all common types of dementia or incidence of cognitive decline		**SBP:** CV- OR 2.32 (95% CI, 0.67-8.08), *P* = 0.19, *I*^2^ = 87% **SBP:** SD- 1.31 (95% CI, 1.03-1.67), *I*^2^ =70% **SBP**: VIM—nil **Diastolic BPV:** nil for all indices	Differed by study, including sex, study center, education, DM, history of vascular diseases, antihypertensive drug at baseline, and mean BP	**Greater BPV associated with cognitive decline**
**He et al (2024)^26^**	Published longitudinal data	Hypertension	Incidence of MCI		HR 1.29 (95 % CI, 0.79-2.09), *I* ^2^ = 73.27	Adjusted for age, sex, education, APOE ε4 genotype, smoking, level of physical, social, or productive activities, race; alcohol use status; BMI; total cholesterol and high-density lipoprotein cholesterol; history of coronary heart disease, heart failure, and stroke	Hypertension not associated with incidence of MCI
**Iseli et al 2019^42^**	Published longitudinal data	OH	For the dichotomous outcome of incidence of cognitive impairment or no	OR 1.19 (95% CI, 1.00-1.42), *P* = 0.048, *I* ^2^ = 58.9%			**OH associated with incidence of cognitive impairment**
**Jia et al 2021^38^**	Published longitudinal data	BPV	Incidence of dementia or cognitive impairment as dichotomous outcome. cognitive decline by change in cognitive test scores per SD increase in BPV	**Visit to visit** **systolic BPV:** pooled HR 1.10 (95% CI, 1.06-1.15), *I*^2^ = 0% Change in cognitive test scores: −0.14 (95% CI, −0.20 to −0.08), *I*^2^ = 0%, when BP increased in a unit of SD Diastolic BPV: **Visit-to-visit diastolic BPV:** −0.17 (95% CI, −0.30 to −0.05) when BP increased in a unit of SD			**Greater BPV associated with cognitive decline**
**Joyce et al 2024** ^28^	Published longitudinal data	Mid-life hypertension	Cognitive domains (memory, attention, executive function, and global cognition), as a continuous outcome, between those with hypertension compared to normotension	Memory: MD = −0.06 (95% CI, −0.20 to 0.08), *I*^2^ = 0% Attention: MD = 0.41 (95% CI, 0.26-0.56), *I*^2^ = 18% Executive function: MD= −0.02 (95% CI, −0.08 to 0.03), *I*^2^ = 36% Global cognition: MD= −0.24 (95% CI, −0.28 to −0.21), *I*^2^ = 12%			**Mid-life hypertension was positively associated with attention, and negatively associated with global cognition**
**Min et al 2020^43^**	Published longitudinal data	OH	OH defined as a sustained reduction in SBP of at least 20 mm Hg and/or DBP decreasing by at least 10 mm Hg for the first 3 minutes of standing or head tilting at least 60°	Worse cognition (HR): 1.18 (95% CI, 1.0-1.35), *I* ^2^ = 69.5%			**OH associated with poorer cognition**
**Ou et al 2020^14^**	Published longitudinal data	Hypertension (SBP ≥130 mm Hg, DBP ≥80 mm Hg, or use of AH medications). Mid-life vs late life hypertension (>65 years)	Global cognition, episodic memory and executive function (Dichotomous)	N/A	Global cognition: RR: 1.55 (95% CI, 1.19-2.03), *I*^2^ = 18% Executive function: 1.22 (95% CI, 1.06-1.41), *I*^2^ = 0% Episodic memory: 1.13 (95% CI, 0.98-1.30), *I*^2^ = 0%	Gender, ethnicity, publication year, educational level, alcohol and tobacco consumption, geographic region, and cerebrovascular or cardiovascular diseases	**Mid-life hypertension associated with poorer global cognition and executive function but not episodic memory**
**Pase et al 2012^41^**	Individual patient data and published data	PWV	Change in MMSE scores—Extracted regression coefficients and CIs were pooled in a meta-analysis along with the regression coefficients and CIs calculated where individual patient data were available		Change in MMSE = −0.03 (95% CI, −0.06 to 0.01)	Age, sex, education, mean arterial pressure, and MMSE scores at baseline	No relationship between PWV and cognitive decline
**Alvarez-Bueno et al 2020^39^**	Published data	PWV	Change in cognitive test scores as a continuous outcome, cross-sectional association: ESs and 95% CIs were calculated for each observed correlation using Cohen’s *d* index and pooled	Global cognition: −0.53 (95% CI, −0.67 to −0.39) Executive function: −0.35 (95% CI, −0.50 to −0.19), Memory: −0.39 (95% CI, −0.70 to −0.09)	Global cognition: −0.21 (95% CI, −0.30 to −0.11) Executive function: −0.08 (95% CI, −0.14 to −0.03) Memory: −0.13 (95% CI, −0.20 to −0.05)	Not specified	**Greater PWV associated with poorer global cognition, executive function, memory**
**Gifford et al 2013** ^31^	Published data, cross-sectional	Hypertension defined by SBP > 140 mm Hg and/or DBP > 90 mm Hg	Pearson’s correlation co-efficient (*r*) was used to identify the strength and direction of the association (continuous): where *r* represents the direction and strength of association between BP (defined for each selected study as SBP or DBP) and cognition, weighted by the sample size of each individual study	Global cognition: *r* = −0.07, 99% CI, −0.13 to −0.02, *P* <0.01 Episodic memory: *r *= −0.18, 99% CI, −0.25 to −0.12, *P* <0.01 Language: *r *= −0.03, 99% CI, −0.18 to 0.12, *P* = 0.62 Executive functioning: *r *= −0.08, 99% CI, −0.25 to 0.09, *P* = 0.21 Attention: *r *= 0.09 (99% CI, −0.01 to 0.19), *P* = 0.02 Information processing speed: *r *= −0.03 (99% CI, − 0.06 to 0.00), *P* = 0.02 Visuo-perceptual abilities: *r *= 0.00 (99% CI, −0.12 to 0.13), *P* = 0.98	Global cognition: *r *= −0.11 (99% CI −0.18 to −0.04), *P* <0.01 Episodic memory: *r *= −0.20 (99% CI = −0.28 to −0.12), *P* <0.01 Language: *r *= −0.22, 99% CI −0.50 to 0.09, *P* = 0.07 Executive functioning: *r *= −0.12, 99% CI −0.34 to 0.12, *P* = 0.20 Attention: *r *= 0.14 (99% CI, 0.03-0.25), *P* = 0.00 Information processing speed: *r *= −0.01 (99% CI, −0.07 to 0.04), *P* = 0.47 Visuo-perceptual abilities: *r *= 0.00 (99% CI, −0.14 to 0.15), *P* = 0.97	Age, education, sex, and vascular factors	Mixed result: **Hypertension associated with poorer global cognition and episodic memory** **Hypertension was associated with enhanced attention performance** No association with language, executive functioning or processing speed
**Huang et al 2024^27^**	Published data	SBP, DBP, hypertension. Different levels of SBP and DBP	For hypertension and DBP, prevalence of cognitive impairment For SBP, incidence of cognitive impairment	Prevalence: OR= 1.53 (95% CI, 1.18 to 1.99), *P* = 0.001 Incident cognitive impairment = 1.13 (95% CI, 1.05, 1.23), *P* = 0.002 SBP <120 mm Hg: 1.15 (95% CI, 1.07-1.25), *P* = <0.001 120-139 mm Hg: 1.26 (95% CI, 1.06-1.49), *P* = 0.010 140-159 mm Hg: 1.15 (95% CI, 1.00-1.32), *P* = 0.05 160-179 mm Hg: 1.02 (95% CI, 1.01-1.04), *P* = 0.009 SBP ≥ 180 mm Hg: 1.17 (95% CI, 0.63-2.17), *P* = 0.62 DBP and prevalence of cognitive impairment = OR 1.38 (95% CI, 1.11-1.72), *P* = 0.004 DBP <80 mm Hg 1.22 (95% CI, 0.95-1.58), *P* = 0.12, 80-99 mm Hg: 1.18 (95% CI, 0.86-1.61), *P* = 0.30 DBP ≥100 mm Hg: 1.96 (95% CI, 1.51-2.56), *P* < .00001			**Hypertension and DBP were associated with the prevalence of CI, SBP was associated with the incidence of CI** **SBP <120 mm Hg, 120-139 mm Hg, 140-159 mm Hg, or 160-179 mm Hg were more likely to have CI, while people with DBP ≥100 mm Hg had higher odds of CI**
**Iseli et al 2019^42^**	Published data	OH	Means and SDs were used for continuous cognitive assessment scale outcomes.	MD −0.51 (95% CI, −0.85 to −0.17), *P* = 0.003, *I*^2^ = 64.9%			**OH associated with poorer cognition**
**Liu 2021^40^**	Published data	Aortic PWV	For cross-sectional studies, Pearson’s *r* correlation coefficients were pooled	Attention: *r* = −0.174 (95% CI, −0.313 to −0.027) Global cognitive function: *r *= −0.122 (95% CI, −0.218 to −0.024) Memory: *r* = −0.061 (95% CI, −0.101 to −0.020) Processing speed: *r *= −0.119 (95% CI, −0.190 to −0.047) MMSE: *r *= −0.11 (95% CI, −0.15 to −0.07)			**Greater PWV associated with poorer global cognition, memory, processing speed**
**Sánchez-Nieto et al 2021^23^**	Published data	Arterial hypertension	All ESs were calculated using standardized MDs, as all studies used a wide variety of scale measures (continuous), group with uncontrolled hypertension compared to the control group	Processing speed: standardized MDs = –0.40 (95% CI, −0.25 to −0.54), *I*^2^ = 28%, *P* = 0.24 Working memory: −0.28 (95% CI, −0.15 to −0.41), *I*2 = 0%, *P* = 0.65 Short-term memory and learning:—0.27 (95% CI, −0.37 to −0.17), *I*^2^ = 0%, *P* = 0.89 Delayed recall: −0.20 (95% CI, −0.35 to −0.05), *I*2 = 0%, *P* = 0.84			**Uncontrolled hypertension associated with poorer cognition: working and short-term memory, delayed recall and processing speed**
**Xue et al 2022^29^**	Published data	Hypertension	Prevalence of MCI	OR 2.44 (95% CI, 1.64-3.62), *P* < .00001			**Hypertension associated with the prevalence of MCI**
**Yang et al 2014^46^**	Published data	Hypertension	Prevalence of vascular cognitive impairment	OR 2.56 (95% CI, 2.03-3.21), *P* = <0.001			**Hypertension associated with the prevalence of vascular cognitive impairment**
**Zhang et al 2023^30^**	Published data	Hypertension	Prevalence of MCI	RR 1.73 (95% CI, 1.59-1.89)			**Hypertension associated with the prevalence of MCI**

Note: The significant findings from each study are highlighted in bold.

Abbreviations: BPV, BP variability; OR, odds ratio; CI, confidence intervals; CV, coefficient of variation; SD, standard deviation; VIM, variation independent of the mean; DM, diabetes mellitus; MCI, mild cognitive impairment; HR, hazard ratio; OH, orthostatic hypotension; MD, mean difference; PWV, pulse wave velocity; MMSE, Mini-Mental State Examination; ES, effect size.

Definitions of hypertension or raised BP varied from SBP ≥130 mm Hg and DBP ≥80 mm Hg,^14^^,^^34^ borderline (140 mm Hg ≤SBP, <160 mm Hg)^22^^,^^33^ up to SBP ≥160 mm Hg or DBP ≥ 90 mm Hg,^18^^,^^36^ SBP >140 mm Hg and/or elevated DBP >90 mm Hg,^31^ with some reviews also including treatment with antihypertensive medication or a history of hypertension as indicating presence of hypertension,^14^^,^^23^^,^^24^^,^^28^^,^^34^ and 10 systematic reviews reporting hypertension only as a binary exposure^3^^,^^20^^,^^21^^,^^25-27^^,^^29^^,^^30^^,^^32^^,^^35^ (Tables 1 and 2). Four reviews included constituent studies that reported on baseline ages in mid-life (<65)^19^^,^^22^^,^^28^^,^^33^, 7 in mid- and late-life,^14^^,^^18^^,^^24^^,^^27^^,^^34-36^ 2 in later life only (>65),^20^^,^^21^ and 6 did not define an age range of their constituent studies.^3^^,^^26^^,^^29^^,^^30^^,^^32^^,^^46^

**Figure 2.**
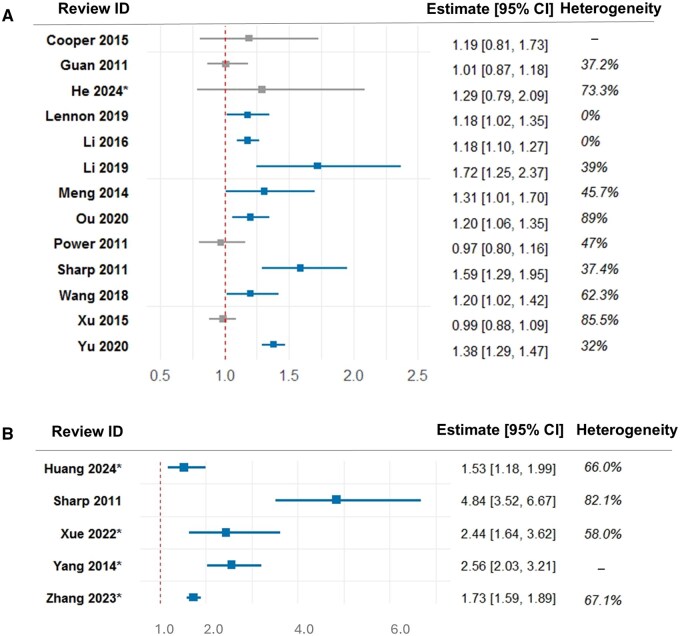
Risk estimates with 95% confidence intervals (CI) for hypertension and cognitive decline* or dementia in (A) longitudinal studies and (B) combined studies.

Overall, 8 out of 13 systematic reviews using longitudinal data reported associations between higher BP and incident dementia;^14^^,^^19^^,^^20^^,^^22^^,^^24^^,^^25^^,^^33^^,^^35^^,^^36^ 3 for all-cause dementia,^14^^,^^33^^,^^35^ 4 for AD,^19^^,^^20^^,^^22^^,^^25^ and 1 for VD^24^ (Figure 2A). One review reported a lower risk of incident dementia with no history of hypertension.^21^ Most found that hypertension or higher SBP was associated with poorer cognitive outcomes, but Meng et al found higher DBP (but not SBP) to be associated with AD.^22^ Reviews that separated mid- and late-life exposures reported greater mid-life SBP to be associated with a greater risk of all-cause dementia.^14^^,^^35^ Additionally, Ou et al^14^ found no association of late-life hypertension and all-cause dementia, while Wang et al^35^ found late-life hypertension or high DBP to be protective for dementia (Table 2).

Five reviews drew neutral conclusions, neither negative nor positive associations between BP and cognitive decline or dementia.^3^^,^^18^^,^^22^^,^^26^^,^^34^ One review on strictly Mendelian randomization studies reported higher BP to be associated with better cognition.^32^

Ou et al reported on the longitudinal association of high BP on cognitive domains and found varied results: Hypertension was significantly associated with worse global cognition (RR 1.55, 95% CI, 1.19-2.03; *I*^2^ = 18%) and executive function (RR 1.22, 95% CI, 1.06-1.41; *I*^2^ = 0%), but not episodic memory (1.13, 95% CI, 0.98-1.30; *I*^2^ = 0%) (Table 3). Joyce et al^28^ reported that hypertension was negatively associated with global cognition but positively with attention.

Systematic reviews which combined longitudinal and cross-sectional data all reported associations between higher BP and higher prevalence of dementia or cognitive decline; 1 for VD^24^ and 4 for cognitive imapairment^27^^,^^29^^,^^30^^,^^46^ (Figure 2B). However, mixed results were found for different domains of cognition: hypertension being associated with poorer global cognition and episodic memory but with enhanced attention performance; and no association with language, executive functioning or processing speed in 1 review.^31^ Poorly controlled hypertension was associated with poorer cognition: working and short-term memory, delayed recall, and processing speed.^23^ Hypertension was also associated with the prevalence of vascular cognitive impairment.^46^  Appendix S4 provides a detailed description of the systematic reviews that reported on hypertension.

#### BP variability

Four systematic reviews reported results for the association between BPV, and dementia or cognitive decline.^14^^,^^37^^,^^38^^,^^45^ Three reviews included meta-analyses of only longitudinal data on incident dementia or cognitive decline separately^14^^,^^37^^,^^38^ (all-cause,^14^^,^^37^^,^^38^ AD,^37^ or VD^37^), while 1 reported longitudinal and cross-sectional associations for combined dementia or cognitive decline^45^ (Tables 2 and 3). Of the various BPV indices, only Chiu et al^37^ specified the inclusion of constituent studies adopting a mixture of office BP, home BP, and ABP measures. In regard to BPV timeframes, reviews included constituent studies with different timeframes, such as short-term/day-to-day to long-term/visit-to-visit BPV.^37^^,^^38^ For BPV indices, coefficient of variation (CV) or standard deviation (SD) were the most frequently used ones for SBP and DBP variability.^37^^,^^38^

All 3 systematic reviews with longitudinal data reported associations between higher BPV and all-cause dementia, and 2 reported similarly for cognitive decline.^37^^,^^38^ All 3 reviews found higher SBP variability to be associated with poorer cognitive outcomes, but Ou et al and Jia et al also found DBP variability to be associated with all-cause dementia or cognitive decline (Tables 2 and 3). Jia et al^38^ also found that both SBP and DBP visit-to-visit and day-to-day variability were associated with dementia and cognitive decline. Chiu et al^37^ demonstrated that BPV measured through either CV or SD was associated with poorer cognitive outcomes. Further details on BPV are provided in Appendix S4.

#### Pulse wave velocity

Pulse wave velocity was used to look at the association of arterial stiffness with dementia or cognitive decline in 3 systematic reviews and meta-analyses.^39-41^ All 3 reviews included meta-analyses of longitudinal data to report on incident dementia or cognitive decline separately,^39-41^ while 2 reported separate cross-sectional associations for dementia or cognitive decline combined^39^^,^^40^ (Tables 2 and 3). Arterial stiffness was measured in the constituent studies of these reviews using carotid-femoral PWV,^39-41^ brachial-ankle PWV,^39^ or aortic PWV.^39^^,^^40^

Two systematic reviews used longitudinal data to report associations of higher PWV and all-cause dementia^40^ or for cognitive decline.^37^^,^^38^ Greater PWV was associated with poorer global cognition,^39^^,^^40^ memory,^39^^,^^40^ executive function,^39^ and processing speed.^40^ One review found no relationship between PWV and cognitive decline.^41^

Two systematic reviews reporting on cross-sectional studies also reported that higher PWV was associated with all-cause dementia,^40^ poorer global cognition,^39^^,^^40^ memory,^39^^,^^40^ executive function,^39^ and processing speed.^40^ Further details are provided in Appendix S4.

#### Orthostatic hypotension

Five systematic reviews reported results for OH related to dementia or cognitive decline.^14^^,^^25^^,^^42-44^ All reviews included meta-analyses of longitudinal data to report on incident dementia^14^^,^^25^^,^^43^^,^^44^ or cognitive decline separately,^14^^,^^42-44^ while 1 also reported the cross-sectional association of OH with cognitive decline^42^ (Tables 2 and 3, Appendix S4). The most widely accepted definition of OH, across the constituent studies of the reviews, was falls of >20 mm Hg SBP and/or >10 mm Hg DBP within the first 3 minutes of standing from a supine position.^14^^,^^25^^,^^42^^,^^44^

All 5 systematic reviews reported associations between higher OH and a greater incidence of all-cause dementia,^14^^,^^43^^,^^44^ AD,^25^ cognitive impairment (dichotomous outcome),^42^ and poorer cognition.^43^ Meta-analysis of cross-sectional data showed that OH was significantly associated with a lower mean MMSE score.^42^

#### Risk of bias and overlap

According to the AMSTAR-2 criteria, 23/31 (74%) reviews were of critically low quality: The remainder were split between high 5/31 (16%), and low 3/31 (10%) quality (supplementary Table S3). All the included reviews provided an appropriate PICO description (population, intervention, control group, and outcome) for organizing the framework of their study question and the eligibility criteria for their selection of studies. Only 10/31 (32%) reviews contained an explicit statement that the methods were established prior to the conduct of the review.

Most of the studies failed to obtain a positive quality assessment because they lacked an established protocol prior to conduct of the review, gave insufficient detail explaining their selection of the study designs for inclusion in the review, did not provide a list of excluded studies with justification, or did not use satisfactory techniques for assessing the risk of bias or publication bias. Moreover, there was considerable heterogeneity in the study characteristics which were not adjusted for, and/or considerable heterogeneity in the study-specific results contributing to meta-analysis. Some reviews did not specify whether cognitive decline was defined using any internationally accepted criteria. The percentage of overlap of constituent studies across the reviews for each BP parameter was for hypertension/SBP/DBP with dementia due to any cause: 6.6%; specific domain of cognition: 2.7%; and cognitive decline or impairment: 0% (supplementary Table S4). The eligibility criteria for reviews focusing on cognitive impairment as the outcome varied widely: Huang et al^27^ included exclusively post-stroke patients, Xue et al^29^ restricted to those with MCI and type 2 diabetes, Yang et al^46^ included only vascular cognitive impairment among Chinese populations, and Zhang et al^30^ restricted to Chinese elderly patients. Overlap of constituent studies among the other parameters was higher: BPV 33.3%, PWV 24.5%, and OH 10.1%.

## Discussion

This umbrella review was designed to facilitate informed understanding and decision-making through the provision of a rigorous summary of the content and quality of the current evidence from systematic reviews and meta-analyses of the relationship between different BP parameters and cognitive decline and/or dementia. Despite some heterogeneity in the evidence base, the weight of the evidence supports an association of higher SBP, DBP, BPV, PWV, and OH on incidence of dementia and cognitive decline, especially for the domains of global cognitive function, executive function, and working memory.

The included reviews support there being a strong association between hypertension, and high SBP or high DBP, with both the incidence and prevalence of cognitive decline or dementia,^14^^,^^19^^,^^20^^,^^22-25^^,^^31^^,^^33^^,^^35^ with various plausible mechanisms being suggested.^7^ Chronic hypertension results in a number of maladaptive and degenerative changes in the cerebral vasculature which may lead to reduced cognitive function. Firstly, hypertension induces atherosclerosis in both extracranial and intracranial arteries supplying the brain.^7^^,^^47^ Hypertension also leads to increased stiffness of large cerebral arteries through hypertrophic and inward remodeling of the cerebral vessels and deposition of collagen and fibronectin and elastin fragmentation of the vessel wall.^7^^,^^48^ In smaller arterioles, hypertension induces endothelial dysfunction and disruption of the blood-brain barrier, triggering inflammatory responses, oxidative stress, and microvasculature injury^7^ and—by altering small penetrating arterioles supplying the subcortical white matter and basal ganglia—promotes small vessel disease with increased risk of lacunar infarction and white matter leasions.^49^ Furthermore, hypertension disrupts cerebral autoregulation, by shifting the autoregulation curve of the cerebral blood flow to the right, allowing a similar level of cerebral perfusion at higher levels of BP, but ischemic brain injury if there are sudden or large reductions in BP.^7^

Reviews which stratified the associations of high SBP/DBP by age provide a clear association between high mid-life BP and the incidence of cognitive decline and dementia. However, there is some evidence that late-life hypertension may have no association or even a “protective” effect.^14^ This reflects previous work highlighting concerns over intense lowering of BP in older adults and suggestions that this group may require a slightly different approach to maintaining optimal BP for adequate cerebral perfusion.^50-54^ It should be noted, however, that associations of lower BP with dementia risk in late life might also indicate impairment in homeostasis, potentially as part of the dementia prodrome by being a marker of cognitive impairment beyond normal biological aging.^55^ Even so, there is now considerable high-quality clinical trial evidence to support the benefits and minimal risks of BP lowering treatment for dementia in the early to mid-phases of later life (mean age 69.1 years).^10^^,^^56^^,^^57^ Our overview of the evidence also revealed an association with poorer function in the domains of global cognitive function, executive function, and memory, although the number of included reviews were small. However, there was heterogeneity around the association with executive function, which is consistent with other studies.^7^ These inconsistencies could be the results of a lack of standardized assessment of cognitive function across studies.^31^ Gifford et al^31^ suggests that this variability in the findings could be due to different tests used in the assessment of executive functioning which might include inhibition or set-shifting measures, cognitive tasks designed to measure 1 domain may be tapping other cognitive processes, increasing variability in findings, test sensitivity, sample selection, and the specific task implemented in each study.

We also found evidence of the relationship of newer BP parameters, raising the potential for future studies to explore the additional risk of these parameters on top of raised SBP/DBP. One of these parameters—BPV—was found to be associated with both cognitive impairment and dementia.^14^^,^^37^^,^^38^ Our summary of the evidence around visit-to-visit systolic BPV shows that higher BPV increases the risk of dementia and increases the risk of cognitive impairment.^38^ While the evidence was strongest for systolic BPV, visit-to-visit diastolic BPV also increased the risk of dementia and cognitive decline, and a meta-regression revealed a linear relationship between higher systolic BPV and risks of dementia and cognitive impairment.^38^ Similar findings were observed in the studies with day-to-day BPV.^38^ Reducing BPV may be a fruitful target to explore for early prevention of dementia.^58^ There are several suggested mechanisms on how higher BPV leads to cognitive impairment. Firstly, repeated BP fluctuations can cause white matter hyperintensities and brain infarctions as shown in magnetic resonance imaging studies,^59^ which can in turn lead to loss of cognitive function.^60^ Higher BPV may also be associated with increased oscillatory shear stress and damage to the vascular endothelium, promoting early atherosclerosis compared to steady blood flow.^61^ This endothelial dysfunction can damage the blood-brain barrier, and impair cerebral autoregulation, leading to loss of cognitive function.^62^

There was also limited evidence for a relationship between PWV and cognition.^63-65^ Higher PWV can cause damage to the brain vasculature and increases the risk of dementia or cognitive decline via several pathological mechanisms. Large arteries lose elasticity with vascular aging, which in turn exposes target organs, including the brain, to higher pulsatile hemodynamic pressure, such as higher blood flow pulsatility.^66^ Excessive blood flow and low resistance to flow in the brain can expose the small arterial vessels to the high-pressure fluctuations, leading to microvascular damage and cognitive decline/dementia.^63^ Small vessels tend to progressively reduce their diameter to counteract these changes, increasing microvascular resistance, and eventually leading to loss of brain function.^65^ Arterial stiffening has also been hypothesized to be associated with tau pathology, β-amyloid plaque deposits, and atrophy of the brain tissue, increasing the risk of MCI or dementia.^67^

Orthostatic hypotension has also been found to be associated with worse cognition.^42^^,^^43^ However, the only 2 reviews in this area included studies that used the MMSE to assess change in cognitive scores. While MMSE is a good screening tool for dementia outcomes, it is less sensitive than other cognitive assessment tools in diagnosing changes in cognition.^68^ Thus, future studies are needed with a consistent battery of neurocognitive tests to improve the estimate of the magnitude of association between OH and cognition. Peters et al^44^ highlight how insufficient data were available from the previously published studies to allow separate meta-analysis for systolic, diastolic, or subclinical OH, which warrants further research. There is some evidence to suggest an underlying mechanism relating OH to increased risk of loss in cognition. Repeated cerebral hypoperfusion due to OH may lead to regional structural changes, such as atherosclerosis, hyperintensities in the deep white matter or leukoaraiosis, and cortical watershed microinfarcts, which may underlie the neurodegeneration process in dementia.^69^^,^^70^ Repeated episodes of hypotension can also lead to cerebral hypoxia and increased production of ß-amyloid protein and neuroinflammation, which in turn can be potentially responsible for cognitive impairment.^71^^,^^72^

### Heterogeneity and risk of bias

There was some evidence of heterogeneity and risk of bias in the included reviews. Variation in the definition of hypertension and/or raised SBP/DBP was present across the reviews, or the definitions were not clearly specified. Gifford et al defined their criteria for hypertension as elevated SBP (>140 mm Hg),^31^ whereas another review defined their criteria as SBP ≥160 mm Hg or DBP ≥90 mm Hg.^18^ One review defined their criteria as SBP 130 mm Hg and DBP 85 mm Hg, or self-report of antihypertensive medication use.^34^ This inconsistency can reduce comparability of the outcomes across different studies. The relationship between high SBP/DBP and cognitive decline and/or dementia is also age-dependent, yet few of the meta-analyses reported estimates for mid- and late-life hypertension. Several reviews also reported on cross-sectional outcomes of dementia and/or cognitive decline, which cannot rule out reverse causality, especially since VD may cause low BP due to loss of sympathetic drive.^73^ Outcomes also varied with some reviews defining their outcome as AD; however, clinical dementia is often a mix of AD and VD, with specific neurodegenerative pathology and cerebrovascular pathology recognized in 61% and 54% of cases, respectively.^74^ Future studies should broaden their criteria to include patients with evidence for mixed causes of dementia, rather than aiming to identify only pure AD or VD, or select only constituent studies that report pure imaging defined pathology to better guide the prevention of dementia. Most studies on variability focused on visit-to-visit variability, which is more useful for measuring long-term variability. Short-term variability measured using 24-hour ABP monitoring has been shown to be useful at detecting subclinical target organ damage and incidence of cardiovascular events, but remains under-researched in dementia.^75^^,^^76^ There are gaps in the populations across the constituent studies in the meta-analyses, such as a lack of data in the oldest old (age ≥75 years) and younger people (<45 years). Constituent studies of the reviews were also predominantly from North America and Europe, with few studies conducted in other countries. Future studies are needed to conclude whether geographical variation can alter the associations between mid- or late-life BP and poorer cognitive outcomes. Finally, there was low overlap in studies for hypertension/SBP/DBP, which was likely due to the differences in selection criteria/process of each review. The reviews encompassing BPV, PWV and OH, showed greater overlap than the others, as their selection criteria were similar.

The strengths of this review are the systematic approach to the search of the evidence, review selection, data extraction and quality assessment, and the use of 2 independent reviewers to reduce potential selection bias. A robust quality assessment was undertaken using internationally recognized criteria, and multiple databases were screened to maximize the inclusion of relevant articles. This review included a range of BP parameters and associated hemodynamic measures. Finally, we assessed the overlap of constituent studies of the included reviews for each BP parameter. There were, however, some limitations. We were unable to reliably perform or reperform any meta-analysis of the individual studies due to the heterogeneity of the data and their reporting; thus, a common point estimate is unavailable. Studies were identified using established databases; however, gray literature was not reviewed, raising the potential for some potential reviews being missed during the screening process.

### Directions for future research

Our overview of the literature reinforces the importance of high BP as a risk factor for cognitive decline and dementia but there is a lack of definitive evidence as to which BP parameter has the strongest association with cognitive decline or dementia across different age groups. Furthermore, as the evidence base emerges for the relationship between nontraditional BP parameters, such as BPV, OH and PWV, and dementia risk, additional research may be required to identify accurate and simple approaches to measure these parameters or risk indicators to gain a greater understanding of their role in cognition. Future cohorts with standardized assessments of BP and cognition and long periods of follow-up across the life course are required to address this research gap. A greater understanding of the emerging BP parameters may indicate potential pathways that, if causal, could indicate a potential novel direction for future risk reduction. Further studies are also needed to consider the effect of short-term BPV on cognitive outcomes using 24-hour ABP monitoring, as well as PP, MAP, and cumulative BP load. There is also a need to study specific risk cohorts, such as those with MCI, to understand which parameters more strongly predict dementia outcomes.

## Conclusion

High BP is associated with worse cognitive outcomes, but the evidence remains scarce regarding which BP thresholds and population groups are the most vulnerable to dementia. Higher BPV, PWV and OH, in addition to hypertension, are also significantly associated with cognitive decline or dementia, and may be useful for risk assessment of cognitive outcomes. Future studies are required to further clarify pathological pathways by which these parameters lead to further cognitive decline or the incidence of dementia, and design novel interventions to address these pathways. For better comparability, studies should use standardized criteria for defining both BP parameters and cognitive outcomes.

## Supplementary Material

hpaf213_Supplementary_Data

## Data Availability

The data extracted for this review can be available upon reasonable request to the authors.
