# Supplementary material for: Associations of Blood Pressure Parameters with Cognitive Decline and Dementia: A Systematic Review of Reviews
Source: Am J Hypertens. 2025 Oct 30;39(4):514–37. doi: 10.1093/ajh/hpaf213 (PMC13017700; doi:10.1093/ajh/hpaf213)
Supplement: hpaf213_Supplementary_Data [file hpaf213_supplementary_data.docx]

# Appendix A: BP Parameter Definitions

SBP and DBP variability defined by using standard deviation (SD) and coefficient of variation (CV, defined as SD/mean); pulse pressure (PP) defined as the difference between SBP and DBP; mean arterial pressure (MAP) defined as the DBP plus one third of the PP; carotid-femoral pulse wave velocity (PWV), which is an indicator of arterial stiffness, defined as PWV=D (meters)/Δt (seconds), where the distance (D) covered by the waves is usually calculated by the surface distance between the two recording sites and the time delay (Δt or transit time) is measured between the feet of the two waveforms at the right common carotid artery and the right femoral artery; orthostatic hypotension (OH) defined as a fall of SBP >20 mmHg and/or a fall of DBP >10 mmHg, from sitting to standing position;^1^ cumulative SBP load, defined as the area-under-curve (AUC) for SBP values ≥130 mmHg divided by the AUC for all measured SBP values over the exposure period.

# Appendix B: Search Strategy

("Blood Pressure" OR "Arterial Pressure" OR "blood pressure monitoring, ambulatory" OR "Hypertension" OR "Pulse Wave Analysis" OR "Carotid-Femoral Pulse Wave Velocity" OR "Blood Pressure" OR "blood pressure variability" OR "mean arterial pressure" OR "pulse pressure" OR "blood pressure load" OR "time in target", "Cognitive Dysfunction" OR "Cognition Disorders" OR "cognition/physiology" OR "cognition/physiopathology" OR "memory disorders/diagnosis" OR "Dementia" OR "dementia, vascular" OR "Alzheimer Disease" OR "cognit*" OR "alzheimer*", and "systematic review" OR "meta-analysis" OR "meta-regression").

# Appendix C: AMSTAR 2 Questions

1. Did the research questions and inclusion criteria for the review include the components of PICO

2. Did the report of the review contain an explicit statement that the review methods were established prior to the conduct of the review and did the report justify any significant deviations from the protocol?

3. Did the review authors explain their selection of the study designs for inclusion in the review?

4. Did the review authors use a comprehensive literature search strategy?

5. Did the review authors perform study selection in duplicate?

6. Did the review authors perform data extraction in duplicate?

7. Did the review authors provide a list of excluded studies and justify the exclusions?

8. Did the review authors describe the included studies in adequate detail?

9. Did the review authors use a satisfactory technique for assessing the risk of bias (RoB) in individual studies that were included in the review?

10. Did the review authors report on the sources of funding for the studies included in the review?

11. If meta-analysis was performed did the review authors use appropriate methods for statistical combination of results?

12. If meta-analysis was performed, did the review authors assess the potential impact of RoB in individual studies on the results of the meta-analysis or other evidence synthesis?

13. Did the review authors account for RoB in individual studies when interpreting/ discussing the results of the review?

14. Did the review authors provide a satisfactory explanation for, and discussion of, any heterogeneity observed in the results of the review?

15. If they performed quantitative synthesis did the review authors carry out an adequate investigation of publication bias (small study bias) and discuss its likely impact on the results of the review?

16. Did the review authors report any potential sources of conflict of interest, including any funding they received for conducting the review?

# Appendix D: Detailed descriptions of the included systematic reviews and meta-analyses

**Hypertension**

Reviews with longitudinal studies

Cooper, Li, Liang, Yang, Yu and Wang et al. did not provide a definition of hypertension or cut off points in their eligibility criteria.^2-7^

Cooper et al. (2015) investigating the association of hypertension and incident dementia in people with MCI did not find a significant association in their unadjusted pooled analysis: OR 1.19 (95% CI 0.81, 1.73).^2^

Guan et al. defined hypertension as having SBP >160 mmHg or DBP ≥90 mmHg).^8^ They used published data from 9 prospective longitudinal studies with follow up ranging from 1 to 32 years and participants aged between 40 to >75 years at baseline.^8^ Guan et al. reported that having SBP >160 mmHg or DBP ≥90 mmHg was associated with developing Alzheimer's Disease using the Mini-Mental State Examination (MMSE); Diagnostic & Statistical Manual-IV (DSM-IV); and the National Institute of Neurological and Communicative Disorders and Stroke-the Alzheimer’s Disease and related Disorders (NINCDS-ADRDA). There were 7,270 and 8,022 subjects with and without hypertension, respectively. After pooling these nine studies, there was no significant difference in the incidence of AD between participants with and without hypertension (RR: 1.01, 95% CI 0.87 - 1.18).

Lee et al.^9^ (2022) used a Mendelian randomisation meta-analysis to look at the associations of risk factors, including SBP and DBP, and compared the results with observational estimates. They used genetic information from genome-wide association studies to look at the

number of single nucleotide polymorphisms, which was used as proxy variables for each risk factor. They also compared with one representative meta-analysis of observational studies for each risk factor. For SBP, they pooled the results from Andrew et al (2021)^10^ and Malik et al. (2021)^11^; pooled estimate 0.91 (95% CI 0.64, 0.99). The pooled results showed high heterogeneity: I^2^= 92.6%. They only found one study looking at DBP (0.89 (95% CI 0.81, 0.98)).

Lennon et al. looked at both systolic hypertension defined by SBP>140 mmHg or SBP>160 mmHg, in separate meta-analyses, and diastolic hypertension using a cut off of > 90mm Hg.^12^ Lennon et al. included seven prospective cohort studies or nested case control studies with mean age ranging from 50.4 to 63 years and follow up ranging from 13 to 22 years. They found that systolic hypertension (>160mm Hg) was associated with higher risk of Alzheimer’s Disease (AD), using the DSM-IV: (HR=1.25, 95% CI 1.06, 1.47, p=0.0065); while systolic hypertension >140mm Hg was also associated with higher risk of AD: HR=1.18, 95% CI 1.02, 1.35, p = 0.021.^12^

Li et al. (2016) reported a published data meta-analysis using 35 cohort studies and found that hypertension was significantly associated with progression to AD of a population with MCI (RR=1.18, 95% CI=1.1 to 1.27, I^2^=0.0%).^3^ Similar results were found by Yu et al. between midlife hypertension and increased risk of AD, RR=1.38 (95% CI 1.29, 1.47),^5^ see Table 3.

Li et al. 2019 reported a published data meta-analysis of seven longitudinal articles with a sample size of 32,222 participants who were aged between 40 and 66 years and with a mean follow up from 12.8 to 40 years.^13^ They reported that high BP (SBP ≥ 160 mmHg) was associated with the incidence of dementia, using DSM-III, DSM-IV, ICD-8, 9 or 10 (medical records); RR 1.72; 95% CI: 1.25-2.37, I^2^ = 39%, p = 0.16.^13^ They also reported that borderline blood pressure (140 ≤ SBP ＜ 160 mmHg) was also associated with dementia incidence (RR 1.41; 95% CI: 1.23-1.62, I^2^ = 10%, p = 0.35).

Liang et al.^4^ (2020) included 9 arms from trials looking at the effect of hypertension on dementia (n = 40,215). However, it was not clear in this article what level of BP was used to define hypertension. Dementia was diagnosed using the ICD-9, DSM-III-R criteria or the NINCDS-ADRDA criteria. Those without hypertension were classified as the exposed group, and those with hypertension as the observation group. They found that those without hypertension had a significantly higher risk of all-cause dementia compared to those with hypertension: OR 0.80 (95% CI 0.65, 0.96).^4^

Meng et al defined hypertension as SBP/ DBP ≥160/95mm Hg.^14^ They included five published prospective studies in their meta-analysis that examined the association between midlife high BP and AD, with mean follow-up ranging from 13.6 years to 37 years, sample size ranging from 1462 to 8006 participants and baseline age ranging from 40 to 68 years. Meta-analysis yielded a random model combined odds ratio, 1.31 (95% CI, 1.01–1.7; z = 2.06, p = 0.039), for the association between high BP and AD risk. AD was mostly diagnosed according

to the National Institute of Neurological and Communicative Diseases and Stroke and the Alzheimer’s Disease and Related Disorders Association (NINCDS-ADRDA) criteria.

Ou et al. defined hypertension according to the seventh report of the Joint National Committee 8th criteria, which defined hypertension as SBP ≥130 mmHg, DBP ≥80 mmHg, or the use of AH medications.^15^ Ou et al. used a published data meta-analysis to combine 136 prospective observational studies, with mean duration of follow-up between 1.5 and 43 years and mean age of participants between 35.3 and 93.2 years. They found that midlife hypertension as defined above was significantly associated with worse global cognition (RR: 1.55 (95% CI, 1.19–2.03), I^2^=18%), as well as worse executive function (RR: 1.22 (95% CI 1.06–1.41) I^2^=0%); see Table 2.^15^ They also found midlife hypertension to be associated with an increased risk of dementia, but heterogeneity was high; RR=1.20 (95% CI 1.06–1.35), I^2^=89%; see Table 3.^15^ The tests used in individual studies to determine changes in cognitive function or diagnostic criteria for dementia were not specified.

Power et al. defined hypertension as 130 mm Hg systolic/85 mm Hg diastolic or self-report of antihypertensive medication use^16^ and conducted a meta-analysis of prospective cohort studies or nested case-control studies conducted in well-defined cohorts; which presented original epidemiologic data on the association between incident Alzheimer disease and measures of blood pressure (BP), diagnosed Alzheimer disease through clinical examination, using defined diagnostic criteria, such as the NINCDS-ADRDA and the Chinese version of the DSMIV. Mean age in years ranged from 53 to 82, while mean follow up ranged from 2 to 27 years, in sample sizes ranging between 406 and 6985 participants. The pooled estimate from the 10 studies reporting on association between a “history of hypertension” and Alzheimer disease was 0.98 (95% CI= 0.80–1.19). Results were similar when the data was combined from studies reporting on “history of hypertension” and “hypertension at enrollment” (summary relative risk RR 0.97, 95% CI 0.80–1.16).

Wang et al. included a meta-analysis of published prospective nested case-control or prospective cohort studies to study the relationship between BP and dementia (all dementia or AD or VD) using DSM – III, DSM – IV, ICD -10 or the NINCD-ADRDA criteria. 21 articles were included in the primary analysis and five articles in the dose-response analysis, with follow-up period of 2.1 to 32 years. Hypertension was defined as SBP ≥140mmHg and/or DBP ≥90mmHg. In the high versus low analyses, pooled results suggested that high SBP was associated with an elevated risk of all dementia (RR: 1.205; 95% CI 1.02-1.42, I² = 62.3%, p = 0.001).^6^ They performed a dose response analysis and found a significant nonlinear association between high SBP and all dementia risk (p for heterogeneity = 0.0717; p for model = 0.0168; p for nonlinearity = 0.0068) was uncovered for population aged 62-82 years. SBP between 110 and 120 mmHg was associated with a lower all dementia risk (three studies were included in this meta-analysis). For population aged 70-86.5 years, a diverse-shaped nonlinear association (p for heterogeneity = 0.2153; p for model = 0.0216; p for nonlinearity = 0.0352) was revealed (three studies were included in this meta-analysis).

Xu et al. defined high SBP as above160mmHg, and low DBP as lower than 70mmHg, looking at longitudinal cohort or retrospective case–control studies.^17^ They did not provide details on individual studies included in the meta-analysis. Their meta-analysis found that SBP ≥160mmHg or low DBP were not associated with AD (combined OR/RR 0.99 (0.88-1.09), I^2^=85.5%, and 1.18 (0.97-1.39), I^2^=4.6% respectively).

Lee et al. used a meta-analysis to examine the associations of multiple risk factors, including SBP and DBP, in two MR studies and compared the results with observational estimates to find that higher SBP was associated with a lower risk of all-cause dementia.

Combined longitudinal and cross-sectional

Sharp et al. defined hypertension based on either a prior diagnosis from the history or a current cross-sectional blood pressure measurement.^18^ Sharp et al. performed a meta-analysis of six longitudinal studies with mean follow up ranging from 3.2 to 10 years and mean age ranging from 57.8 to 90 years. They showed that hypertension was significantly associated with increased risk of incident vascular dementia (odds ratio, OR: 1.59, CI 1.29, 1.95, p<0.0001).^18^ A similar association between hypertension and the risk of prevalent vascular dementia was found in the five cross-sectional studies (OR: 4.84, 95% CI: 3.52, 6.67, p<0.00001), Table 3.^18^

Gifford et al. defined hypertension as having SBP>140 mmHg and/or DBP>90 mmHg.^19^ Pearson’s correlation co-efficient was used to identify the strength and direction of the association. They reported correlations limiting analyses to eight studies that statistically adjusted for demographic variables or vascular factors. Global cognition score was measured using a composite score or MMSE; and episodic memory was assessed via the California Verbal Learning Test-II, Consortium to Establish a Registry for Alzheimer’s Disease (CERAD) Word List Immediate and Delay Recall, or the Wechsler Memory Scale (WMS), see Table 1. Tests used to assess language, attention, executive functioning, information processing speed, visuoperceptual skills are summarised in Table 1.^19^ Gifford et al. found higher blood pressure to be correlated with lower global cognition (r = -0.11, p < .001, 99% CI = -0.18 to -0.04), where r represents the direction and strength of association between SBP/DBP and cognition, weighted by the sample size of each individual study, and episodic memory (r = -0.20, p < .001, 99% CI = -0.28 to -0.12).^19^ They found no correlation with language (r = -0.22, p = 0.07, 99% CI = -0.50 to 0.09), executive functioning (r = -0.12, p = 0.20, 99% CI = -0.34 to 0.12), information processing speed (r = -0.01, p = .47, 99% CI = -0.07 to 0.04), or visuo-perceptual abilities (r = 0.00, p = .97, 99% CI = -0.14 to 0.15). They found a positive association between higher BP and better attention (r =0.14, p = 0.002, 99% CI = 0.03 to 0.25).

Sánchez-Nieto et al. included published data from 8 studies and defined arterial hypertension as those who were given a medical diagnosis of hypertension or by being prescribed medications for high blood pressure.^20^ It was not specified whether they included only cross-sectional or prospective studies in their meta-analysis. They found that the group with uncontrolled hypertension (resting systolic BP>179 mmHg or diastolic BP>105 mmHg) compared to the control group had lower performance in: processing speed, standardised mean differences (SMD) = 0.40 (95% CI: 0.25-0.54, I^2^ = 28%; p for heterogeneity = 0.24); working memory, SMD = 0.28 (95% CI: 0.15-0.41; I^2^ = 0%; p = 0.65); short-term memory and learning SMD = -0.27 (95% CI: -0.37 – -0.17; I^2^ = 0%; p = 0.89), and delayed recall SMD = -0.20 (95% CI: -0.35, -0.05; I^2^ = 0%; p = 0.84), see Table 3.^20^

Yang et al. (2014) investigating the association of hypertension and prevalence of vascular cognitive impairment in 42 studies including 3,282 cases and 7,815 controls found a significant association; OR 2.56, 95% CI 2.03–3.21, p= <0.05.^7^

De Heus et al. investigated the combined outcome of dementia or cognitive impairment and found an association between mean systolic BP and cognitive decline/dementia, average age 73±7 years. They analysed a mix of cohort, nested case controls, RCTs analysed as a cohort and cross-sectional studies. Higher mean SBP was associated with dementia/cognitive impairment (OR=1.12 (95% CI 1.02, 1.29]; I^2^=82%).^21^ However, they did not provide the unit of change in SBP which drove this change. Similar results were found for mean diastolic BP (OR=1.16 (95% CI 1.04, 1.29]; I^2^=3%). DSM-IV criteria, MMSE <24 at follow-up, Digit Symbol Substitution Test, Mini-Cog ≤2, NINCDS-ADRDA, DSM-III-R criteria, prescription of antidementia drugs or dementia admission, or ICD codes/prescription were used as criteria for diagnosis of dementia/cognitive impairment.

**Detailed descriptions of the included systematic reviews for blood pressure variability (BPV)**

The systematic reviews drew constituent studies predominantly from North American, European and Chinese populations, with some studies including Australian, Japanese, Taiwanese and Korean populations. Chiu et al. used a published data meta-analysis to combine eight longitudinal studies with 7,924,168 participants over a follow up duration of 3 months to 22 years (mean age range: 54.3–84.4 years).^22^ All reviews included constituent studies that reported on baseline ages in both mid- and late-life, ranging from 35.3 to 93.2 years. Any measure of SBPV or DBPV obtained from ambulatory, home, or visit-to-visit BP monitoring was considered eligible. The BPV indices included the following three categories: overall variability, variability between consecutive visits, and the extremes in values on a single visit. Overall variability was assessed using standard deviation (SD), coefficient of variation (CV), and variance independent of the mean (VIM).^22^ Incidence of all common types of dementia, including Alzheimer’s disease and vascular dementia, verified by licensed physicians or related professionals or medical records was the primary outcome. The secondary outcome was the incidence of cognitive decline across any period, obtained by standardised neuropsychological tests, including Mini-Mental State Examination (MMSE), Montreal Cognitive Assessment (MoCA), Cambridge Cognition Examination (CAMCOG), etc., at least twice.^22^ All studies were measured using either mid-term or long-term BPV. SBP-CV and SBP-SD showed significant positive associations with all-cause dementia risk (SBP-CV: HR = 1.45 [95% CI, 1.11–1.90] I^2^ = 78%; SBP-SD: HR = 1.31 [95% CI, 1.03–1.67] I^2^ =70%), whereas SBP-VIM and DBP-CV demonstrated no significant association.^22^

Jia et al. conducted a meta-analysis of 14 prospective and two retrospective cohort studies. The median age of the study participants was from 50.9 to 79.9 years. Study participants were mainly from community-dwelling population except for 2 studies with recruitment from a factory and a database of an insurance service. Thirteen studies evaluated visit-to-visit BPV with a median follow-up period up to 14 years; whereas 3 studies evaluated day-to-day BPV with a median follow-up period up to 7.8 years. Standard deviation was the most common measurement of BPV. Visit-to-visit systolic BPV was significantly associated with cognitive impairment: pooled HR=1.10 (1.06–1.15), I^2^=0%; participants with an increased level of visit-to-visit SBPV had more cognitive decline, that is, cognitive test scores changed by −0.14 (95% CI −0.20, −0.08, I^2^=0%) when BP increased in a unit of SD.^23^ A cutoff of MMSE score ≤24, MoCA <26 or a decline >1.5 standard variation was used to measure the incidences of cognitive impairment. The change in the MMSE or MoCA score during the follow up period was defined as the cognitive decline. Standardised coefficients were calculated from linear regression coefficients i.e. the coefficient per SD increased in BPV. Visit-to-visit diastolic BPV also showed an increased risk of cognitive decline, cognitive tests scores changed by −0.17 (95% CI −0.30, −0.05) when BP increased in a unit of SD.^23^

They also looked at the association of visit-to-visit vs day-to-day SBPV with dementia as the outcome diagnosed according to the DSM- III or IV, or the criteria of the NINCDS-ADRDA or antidementia drugs prescribed at least 2× and the codes with Alzheimer disease, and found that visit-to-visit SBPV was associated with dementia incidence, HR=1.11 (95% CI 1.05–1.17, I^2^=53%), but the association was slightly stronger for the day-to-day SBPV, HR=1.38 (95% CI 1.23–1.55). Similar results were found for diastolic BPV (DBPV); visit-to-visit HR=1.14 (95% CI 1.04 to 1.25) vs. day-to-day HR=1.38 (95% CI 1.23–1.55).^23^

Ou et al. used a published meta-analysis to combine 136 prospective observational studies, details of which has been described above. Excessive DBP change (≥5 mm Hg) in midlife was excess risk of dementia, RR=1.65 (95% CI, 1.28–2.11), I^2^=0%.^15^ In late-life, both excessive systolic and diastolic BPV were associated with higher dementia risk, RR=1.99 (95% CI 1.46–2.29), I^2^=0%, and RR=2.09 (95% CI 1.27–3.44), I^2^=57%, respectively.^15^

De Heus et al. (2021) reported that higher systolic BPV was significantly associated with an increase in the combined outcome of dementia or cognitive impairment, but with high heterogeneity: OR=1.25 (95% CI 1.16, 1.35); I^2^=87%.^24^ Similar results were found for diastolic BPV (OR=1.20 (95% CI 1.12, 1.29]; I^2^=83%.

**Detailed descriptions of the included systematic reviews for PWV**

The systematic reviews drew constituent studies predominantly from North American and European, with some studies including Australian, Brazilian and Singaporean populations. Mean or median PWV ranged from 4.96 to 14.3 m/s. All reviews included constituent studies that reported on baseline ages in both mid- and late-life, overall ranging from 46 to 87 years. Liu et al. did a meta-analysis of 29 published articles, except one, all the other studies used cfPWV and showed mean or median PWV ranging from 4.96 to 14.3 m/s. Eighteen studies with 15,489 participants were eligible for meta-analysis of association between aortic PWV and cognitive function. They showed associations between aortic PWV and attention (r = −0.174, 95% CI −0.313, −0.027), global cognitive function (r = −0.122, 95% CI −0.218, −0.024), memory (r = −0.061, 95% CI −0.101, −0.020), and processing speed (r = −0.119, 95% CI −0.190, −0.047); aortic PWV and Mini-Mental State Examination (MMSE) (r = −0.11, 95% CI −0.15 to −0.07). Higher aortic stiffness predicted lower MMSE scores within the sample (b = -0.03, 95% confidence interval (CI): -0.06 to 0.01, *n* = 3947), although studies were not all homogeneous, and statistical heterogeneity was present (I^2^ = 71.9%, p= 0.01).^25, 26^ They also looked at PWV and dementia, and found that participants within the highest category of aortic PWV had higher odds of dementia compared to the lowest category of PWV; OR=2.10 (95% CI 1.16, 3.80), I^2^=64.7%, p=0.06.^25^

Pase et al. included four longitudinal studies for meta-analysis conducted over an average of 5 years follow up.^26^ The most widely used measure of aortic stiffness was cf-PWV, which was implemented in all but one of the reviewed studies (cardio–ankle vascular index). Across studies, there were sufficient data to examine the association between aortic stiffness (cf-PWV) and cognitive decline (end-point MMSE scores) in meta-analysis. Of the four relevant studies, the raw individual patient data were obtained for two, while aggregate statistics were obtained from the other two. Across all four studies, higher aortic stiffness was found to predict lower MMSE scores within the sample (b =-0.03, 95% CI: 0.06 to 0.01, n=3947).^26^

Alvarez-Bueno et al. conducted a meta-analysis of 29 cross-sectional and 9 longitudinal published studies with mean age ranging from 46.0 to 85.0 years, mean SBP ranging from 116.0 to 159.0 mm Hg; and mean DBP ranging from 64.0 to 90.3 mm Hg.^27^ PWV was measured using carotid-femoral (cfPWV) procedures in all studies, but four used brachial-ankle (baPWV) and one used aortic PWV. The reported mean PWV ranged from 4.9 to 6.9 m/s for cfPWV and from 15.3 to 23.7 for baPWV.^27^ Pooled results showed a negative cross-sectional association between PWV and cognition: −0.21 (95% CI −0.30 to −0.11) for global cognition, −0.08 (95% CI −0.14 to −0.03) for executive function, and −0.13 (95% CI −0.20 to −0.05) for memory.^27^ Tests used to measure cognitive function are detailed in Table 2. The pooled values for the longitudinal association of PWV and global cognition, executive function and memory were −0.21 (95% CI, −0.36 to −0.06), −0.12 (95% CI, −0.22 to −0.02), and −0.05 (95% CI, −0.12 to 0.03), respectively.^27^

**Detailed descriptions of the included systematic reviews for OH**

Min et al. included 13 prospective studies that defined OH as ^28^ In total, 13 studies reported worse cognition: 6 for dementia, 5 for cognitive impairment (CI), and 2 for cognitive decline (CD). Six studies, consisting of 48,179 participants, referred to dementia. The diagnostic criteria were various among the included studies.^28^ One study ascertained dementia based on ICD-9, two other studies by DSM-III-R, and the remaining three studies diagnosed dementia based on DSM-IV. Five studies that included a total of 5829 participants reported data concerning CI. Of the five CI studies, CI was defined as MMSE <=23 in one study, as a score of 0 or 1 on the three words delayed recall task of the MMSE in another and as MMSE <=24 in the remaining three. Additionally, two studies that included 2845 participants also reported results about CD. One study defined CD as a drop in MMSE score of >=1 point, and the other study defined CD as a MMSE score drop of >=3 points. Follow-up ranged from 2 to 28 years and mean age ranged from 45 to 83.5 years. OH was found to be significantly associated with worse cognition (combined dementia, CI and CD): - HR 1.18 with 95% CI: 1.03–1.35, I^2^ = 69.5%,; and dementia; HR=1.30 (95% CI 1.143, 1.48), I^2^ = 31%.^28^

Peters et al. conducted an individual patient data meta-analysis on 5 prospective longitudinal studies, with mean follow up ranging from one/two years to 28 years and mean age ranging from 45 to 73.4 years.^29^ The definition of OH in the included studies varied, for example, one study defined OH as the fall of SBP >20 mmHg or DBP >10mmHg from supine position, after standing for 1-10 minutes, or a fall of SBP >40 mmHg or DBP >20 mmHg immediately after standing (Swedish Good Aging in Skåne Study. Elmståhl et al 2014). Another study defined OH as change from supine to standing at 1, 2 and 3 minutes after standing and a fall of SBP >=20mmHg or DBP >=10mmHg within 3 minutes (Rotterdam Study. Wolters et al 2016). Peters et al. found that OH was associated with higher RR; pooled RR 1.21 (95% CI 1.09–1.35) for fixed effects analysis, with low heterogeneity I^2^=10.4%, and no significant publication bias (Egger bias P=0.1463). ^29^

Iseli et al. included 32 studies (22 cross-sectional; 10 longitudinal) reporting data of 28,980

individuals with mean age ≥65 years. OH was defined as a decrease of at least 20 mmHg SBP and/or 10 mmHg DBP within the first 3 min of standing. Prevalence of OH ranged from 3.3% to 58%. Of the 32 studies, 18 reported an association between OH and worse cognitive performance and 14 reported no association. MMSE was the most commonly used cognitive assessment tool. Means and standard deviations (SD) were used for continuous cognitive assessment scale outcomes. For the dichotomous outcome of diagnosis of cognitive impairment, the odds ratio (OR) for reported prevalence in the groups with and without OH was used. Orthostatic hypotension (OH) was significantly associated with a lower mean MMSE score, mean difference= −0.51 (95% CI: −0.85, −0.17, p=0.003), I^2^=64.9%.^30^ They also reported on longitudinal studies reporting odds ratios for cognitive impairment in populations with OH compared to those without OH. There was an association between OH and cognitive impairment in older adults on longitudinal data [OR (95% CI): 1.19 (1.00, 1.42), p=0.048], with high heterogeneity (I^2^=58.9%).
Yu et al. reported on 104 modifiable factors from 243 observational prospective studies, including hypertension in midlife and orthostatic hypotension.^5^ Details on the individual studies reporting hypertension were not specified. They found a significant association between OH and Alzheimer’s Disease, RR= 1.18 (95% CI 1.02-1.35).^5^

**Supplementary Table S1: List of excluded studies after full text screening with reasons for exclusion**

| **Author** | **Title** | **Reason for Exclusion** |
| --- | --- | --- |
| Downer M, McColl A, Li L, Pendlebury ST, Rothwell P | The influence of prior comorbidity and multimorbidity on the risk of post-stroke dementia: A meta-analysis and population-based study | Full text not found* (Article with this title was not found for full text screening) |
| Aimagambetova 2024 | Arterial stiffness measured by pulse wave velocity correlated with cognitive decline in hypertensive individuals: a systematic review | No meta-analysis |
| Appiah KOB, Patel M, Panerai RB, Robinson TG, Haunton VJ | Increased blood pressure variability following acute stroke is associated with poor long-term outcomes: a systematic review | No meta-analysis |
| Blanken AE, Nation DA | Does Gender Influence the Relationship Between High Blood Pressure and Dementia? Highlighting Areas for Further Investigation | No meta-analysis |
| Blom 2013 | The influence of vascular risk factors on cognitive decline in patients with dementia: A systematic review | No meta-analysis |
| Bosi 2022 | Environmental and lifestyle risk factors for early-onset dementia: a systematic review | No meta-analysis |
| C. A. Feldstein | Association between chronic blood pressure changes and development of Alzheimer’s disease | No meta-analysis |
| Desai 2024 | Evidence for causal links between known modifiable risk factors and dementia: A systematic review of Mendelian randomization studies | No meta-analysis |
| El-Metwally A, Toivola P, Al-Rashidi M, Nooruddin S, Jawed M, Alkanhal R, et al | Epidemiology of Alzheimer's Disease and Dementia in Arab Countries: A Systematic Review | No meta-analysis |
| Forte 2019 | Effects of Blood Pressure on Cognitive Performance: A Systematic Review | No meta-analysis |
| Forte 2020 | Effects of Blood Pressure on Cognitive Performance in Aging: A Systematic Review | No meta-analysis |
| Gibson 2023 | The association between 24-hour blood pressure profiles and dementia | No meta-analysis |
| Gowda 2025 | The impact of chronic diseases on cognitive impairment in rural population of India: A focus on diabetes, hypertension, cardiovascular disease, and stroke | No meta-analysis |
| Hadjiev D, Mineva P | Hypertension, vascular cognitive disorders and neuroprotection | No meta-analysis |
| Haghayegh 2025 | Critical Review of the Methodological Shortcoming of Ambulatory Blood Pressure Monitoring and Cognitive Function Studies | No meta-analysis |
| Hamrah 2023 | Modifiable Risk Factors for Dementia Among Migrants, Refugees and Asylum Seekers in Australia: A Systematic Review | No meta-analysis |
| Khan 2024 | The Impact of Hypertension on Cognitive Decline and Alzheimer's Disease and Its Management: A Systematic Review | No meta-analysis |
| Kloppenborg 2008 | Diabetes and other vascular risk factors for dementia: Which factor matters most? A systematic review | No meta-analysis |
| Kudo T, Imaizumi K, Tanimukai H, Katayama T, Sato N, Nakamura Y, et al | Are cerebrovascular factors involved in Alzheimer's disease? | No meta-analysis |
| Kuzma 2018 | Which Risk Factors Causally Influence Dementia? A Systematic Review of Mendelian Randomization Studies | No meta-analysis |
| Lancaster 2022 | Blood pressure change and cognition in childhood and early adulthood: a systematic review | No meta-analysis |
| Lancaster 2022 | Blood pressure change and cognition in childhood and early adulthood: a systematic review | No meta-analysis |
| Li 2017 | Arterial stiffness and cognitive impairment | No meta-analysis |
| Li, Xiaoxuan Lyu, P, Ren, Y, An, J, Dong, Y | Arterial stiffness and cognitive impairment | No meta-analysis |
| Lucas 2022 | Knowledge gaps and future directions in cognitive functions in children and adolescents with primary arterial hypertension: A systematic review | No meta-analysis |
| Moraes 2019 | Executive function in systemic arterial hypertension | No meta-analysis |
| Nagai 2017 | Visit-to-Visit Blood Pressure Variability and Alzheimer’s Disease: Links and Risks | No meta-analysis |
| Nguyen 2023 | Risk, protective, and biomarkers of dementia in Indigenous peoples: A systematic review | No meta-analysis |
| Patterson 2008 | Diagnosis and treatment of dementia: 1. Risk assessment and primary prevention of Alzheimer disease | No meta-analysis |
| Peters 2020 | Trajectory of blood pressure, body mass index, cholesterol and incident dementia: systematic review | No meta-analysis |
| Polidori 2012 | A Review of the Major Vascular Risk Factors Related to Alzheimer’s Disease | No meta-analysis |
| Purnell 2009 | Cardiovascular risk factors and incident Alzheimer disease: A systematic review of the literature | No meta-analysis |
| Qiu 2011 | Epidemiological findings of vascular risk factors in Alzheimer’s disease: implications for therapeutic and preventive intervention | No meta-analysis |
| Rabkin, S. W. | Arterial stiffness: Detection and consequences in cognitive impairment and dementia of the elderly | No meta-analysis |
| Singer 2014 | Arterial stiffness, the brain and cognition: A systematic review | No meta-analysis |
| Surawan 2017 | to evaluate the risk factors for prognostic of post-stroke dementia (PSD) | No meta-analysis |
| van den Berg 2009 | Type 2 diabetes mellitus, hypertension, dyslipidemia and obesity: A systematic comparison of their impact on cognition | No meta-analysis |
| van Sloten 2015 | Association between arterial stiffness, cerebral small vessel disease and cognitive impairment: a systematic review and meta-analysis | No meta-analysis |
| Walker 2020 | Alzheimer’s Disease and Related Dementia in Indigenous Populations: A Systematic Review of Risk Factors | No meta-analysis |
| Walker 2020 | Alzheimer's Disease and Related Dementia in Indigenous Populations: A Systematic Review of Risk Factors | No meta-analysis |
| Xie 2024 | Prevalence and risk factors of cognitive impairment in Chinese patients with hypertension: A systematic review and meta-analysis | No unexposed group |
| Cations M, Withall A, Low LF, Draper B | Can exposure to environmental and lifestyle risk factors bring forward the age of dementia onset? | Only abstract |
| Duschek S, Schandry R | Subjektive Beschwerden und kognitive Minderleistungen bei essentieller Hypotonie [Subjective Symptoms and Cognitive Deficits in Essential Hypotenstion] | Only abstract |
| Isik AT, Erken N, Yavuz I, Kaya D, Ontan MS, Ates Bulut E, et al | Orthostatic hypotension in patients with Alzheimer's disease: a meta-analysis of prospective studies | Only abstract |
| Kuzma E, Hannon E, Zhou A, Lourida I, Bethel A, Levine D, et al | A systematic review of mendelian randomization studies investigating causal associations between risk factors and dementia | Only abstract |
| Lo JW, Crawford JD, Desmond DW, Akinyemi RO, Bae HJ, Bordet R, et al | The relationship of hypertension, diabetes and othervascular risk factors with post stroke cognitive function: The strokog (stroke and cognition) consortium | Only abstract |
| Lo JW, Crawford JD, Desmond DW, Godefroy O, Jokinen H, Mahinrad S, et al | Profile of and Risk Factors for Post-Stroke Cognitive Impairment in Diverse Ethno-Regional Groups | Only abstract |
| Low LF, Wai HY, Brodaty H | Does apolipoprotein E genotype interact with the effects of potentially modifiable mid- and late-life risk factors for dementia? A systematic review | Only abstract |
| Pendlebury ST, Rothwell PM | Predictors of post-stroke dementia: A quantitative systematic review and meta-analysis | Only abstract |
| Szoeke C, Goodwill AM | Sex Differences in Vascular Risk Factors and Dementia: A Systematic Review and Meta-Analysis | Only abstract |
| Wang A, Chong E, Gupta A | Abnormal Dipping Pattern of Nocturnal Blood Pressure as a Predictor of Cognitive Impairment and/ or Dementia: A Systematic Review and Meta-Analysis | Only abstract |
| Waziry R, Claus J, Hofman A | Dementia risk following ischemic stroke: A systematic review and meta-analysis of factors collected at time of stroke diagnosis | Only abstract |
| Xin L, Zhao X, Yu J | Systematic review of the effect of blood pressure variation on cognitive decline in the elderly with hypertension | Only abstract |
| Xu W | The construction of evidence-based profile for Alzheimer's prevention: A meta-analysis and systematic review assessing the current evidence based on prospective observational studies and randomized controlled trials | Only abstract |
| Xu W | Blood pressure and the risk of dementia: A systematic review and dose-response metaanalysis of prospective studies | Only abstract |
| Zhao X, Lin X, Kou C, Wang Q, Yu J | Blood Pressure Variability and Mind Cognition Impairment: A Systemic Review and Meta-Analysis | Only abstract |
| Batty GD, Russ TC, Starr JM, Stamatakis E, Kivimäki M | Modifiable cardiovascular disease risk factors as predictors of dementia death: pooling of ten general population-based cohort studies | Outcome not relevant |
| Ottaviani C, Thayer JF, Verkuil B, Lonigro A, Medea B, Couyoumdjian A, et al | Physiological concomitants of perseverative cognition: A systematic review and meta-analysis | Outcome not relevant |
| Pasdar 2023 | Orthostatic hypertension and major adverse events: a systematic review and meta-analysis | Outcome not relevant |
| Senff 2025 | Modifiable risk factors for stroke, dementia and late-life depression: a systematic review and DALY-weighted risk factors for a composite outcome | Outcome not relevant |
| Velazquez N, Coetzee O | Vascular dementia lifestyle and nutrition prevention strategies: | Outcome not relevant |
| Xue H, Hou P, Li Y, Mao X, Wu L, Liu Y | Factors for predicting reversion from mild cognitive impairment to normal cognition: A meta-analysis | Outcome not relevant |
| Abate G, Zito M, Ferrari-Ramondo V, Di Iorio A | Blood pressure and dementia: A review | Systematic search not described |
| Anderson C, Teo K, Gao P, Arima H, Dans A, Unger T, et al | Renin-angiotensin system blockade and cognitive function in patients at high risk of cardiovascular disease: analysis of data from the ONTARGET and TRANSCEND studies | Systematic search not described |
| Birkenhäger WH, Staessen JA | Progress in cardiovascular diseases: cognitive function in essential hypertension | Systematic search not described |
| Bogaerts 2022 | Disentangling the varying associations between systolic blood pressure and health outcomes in the very old: an individual patient data meta-analysis | Systematic search not described |
| Bogaerts JMK, Poortvliet RKE, van der Klei V, Achterberg WP, Blom JW, Teh R, et al | Disentangling the varying associations between systolic blood pressure and health outcomes in the very old: an individual patient data meta-analysis | Systematic search not described |
| C. A. Feldstein | Effects of blood pressure changes on Alzheimer’s disease | Systematic search not described |
| Carmichael O | Preventing vascular effects on brain injury and cognition late in life: Knowns and unknowns | Systematic search not described |
| Duron E, Hanon O | Hypertension, cognitive decline and dementia | Systematic search not described |
| Fenclová E, Albrecht J, Harsa P, Jirák R | Rizikové faktory Alzheimerovy nemoci [Risk factors for Alzheimer’s disease] | Systematic search not described |
| Hanon O | [Hypertension and dementia] | Systematic search not described |
| Hanon O, Forette F | Treatment of hypertension and prevention of dementia | Systematic search not described |
| Igase M, Kohara K, Miki T | The association between blood pressure and cognitive function | Systematic search not described |
| King HE, Miller RE | Hypertension: Cognitive and behavioral considerations | Systematic search not described |
| Kivipelto M, Laakso MP, Tuomilehto J, Nissinen A, Soininen H | Hypertension and hypercholesterolaemia as risk factors for Alzheimer's disease: Potential for pharmacological intervention | Systematic search not described |
| Launer LJ | The epidemiologic study of dementia: A life-long quest? | Systematic search not described |
| Lennon 2023 | Use of Antihypertensives, Blood Pressure, and Estimated Risk of Dementia in Late Life: An Individual Participant Data Meta-Analysis | Systematic search not described |
| Leung 2023 | Cognition, function, and prevalent dementia in centenarians and near-centenarians: An individual participant data (IPD) meta-analysis of 18 studies | Systematic search not described |
| Li C, Ma Y, Hua R, Yang Z, Zhong B, Wang H, et al | Dose-Response Relationship Between Long-Term Blood Pressure Variability and Cognitive Decline | Systematic search not described |
| Mossello E | Hypertension, hypotension, longevity and dementia | Systematic search not described |
| Nelson L, Gard P, Tabet N | Hypertension and inflammation in Alzheimer’s disease: Close partners in disease development and progression! | Systematic search not described |
| Palta, P, Albert, M. S., Gottesman, R. F. | Heart health meets cognitive health: Evidence on the role of blood pressure | Systematic search not described |
| Panza F, D' Introno A, Colacicco AM, Capurso C, Del Parigi A, Capurso SA, et al | Cognitive frailty: Predementia syndrome and vascular risk factors | Systematic search not described |
| Pase MP | Modifiable vascular markers for cognitive decline and dementia: The importance of arterial aging and hemodynamic factors | Systematic search not described |
| Qiu C, De Ronchi D, Fratiglioni L | The epidemiology of the dementias: An update | Systematic search not described |
| Qiu, C, Xu, W, Fratiglioni, L | Vascular and psychosocial factors in Alzheimer’s disease: Epidemiological evidence toward intervention | Systematic search not described |
| Richard E, van Charante EPM, van Gool WA | Vascular risk factors as treatment target to prevent cognitive decline | Systematic search not described |
| Rinn WE | Mental decline in normal aging: A review | Systematic search not described |
| Rodrigue KM, Bischof GN, Cabeza R, Nyberg L, Park DC | The modifying role of hypertension in cognitive and brain aging | Systematic search not described |
| Scullin MK, Le DT, Shelton JT | Healthy heart, healthy brain: Hypertension affects cognitive functioning in older age | Systematic search not described |
| Serrano-Pozo, A, Growdon, JH. | Is Alzheimer’s disease risk modifiable? | Systematic search not described |
| Silvestrini M, Viticchi G, Altamura C, Luzzi S, Balucani C, Vernieri F | Cerebrovascular assessment for the risk prediction of Alzheimer's disease | Systematic search not described |
| Tolppanen, A, Solomon, A, Soininen, H, Kivipelto, M | Midlife vascular risk factors and Alzheimer's disease: Evidence from epidemiological studies | Systematic search not described |
| Waldstein SR, Katzel LI, Waldstein SR, Elias MF | Hypertension and cognitive function | Systematic search not described |

**Supplementary Table S2: Geographical distribution of constituent studies of included reviews**

| **Review ID** | **Geographical distribution** |
| --- | --- |
| **Alvarez-Bueno 2020** | USA (10), Europe (14), Japan, Greece (2), Korea (2), Singapore, Brazil, Australia |
| **Chiu 2021** | China (3), South Korea (2), France, USA, Japan |
| **Cooper 2015** | Europe (3), Iran |
| **de Heus 2021** | Japan (7), Erupe (7), Multinational (2), Taiwan, South Korea, USA |
| **Gifford 2013** | USA (7), Europe(3), Taiwan, Hong Kong |
| **Guan 2011** | USA (4), Sweden (2), Canada (2), Japanese American |
| **He 2024** | USA (2), Singapore |
| **Huang 2024** | China (5), Europe (5), Australia, Ghana |
| **Iseli 2019** | Europe (10), USA (3), Japan, China, Israel |
| **Jia 2021** | Europe (2), USA, Australia, Japan, Korea, China, and Taiwan |
| **Joyce 2024** | USA (7), Europe (5), Multinational (2), Asian |
| **Lee 2022** | Europe (4), USA |
| **Lennon 2019** | USA (2), Europe (2), Taiwan, Korea, Japan |
| **Li 2016** | Europe (5), China, Israel |
| **Li 2019** | Europe (3), Japan (2), USA (2), |
| **Liang 2020** | USA (2), Taiwan |
| **Liu 2021** | Europe (16), USA (6), Australia (2), South Korea, China, Malaysia, Pakistan, Iran |
| **Meng 2014** | Europe (3), Japanese American, Korean, USA, Japan |
| **Min 2020** | Europe (12), USA (3), China (5) |
| **Ou 2020** | North America (40-45), Aisa (35-40), Europe (35-40), Australia (5), Africa (3-5), South America (2-3), Middle East (2-3), Multinational (5) |
| **Pase 2012** | Europe (3), USA (2), Japan |
| **Peters 2018** | Europe (4), Multinational (1) |
| **Power 2011** | Europe (6), North America (10), Nigeria, Taiwan |
| **Sánchez-Nieto 2021** | Not specified |
| **Sharp 2011** | Longitudinal studies: US or Canada (5), and one in Taiwan Cross-sectional - Israel, Arabs, Spain, Turkey, Taiwan, Italy |
| **Wang 2018** | USA (11), European (10), Asian (3), African (1) |
| **Xu 2015** | USA (4), Europe (3), China (2), Taiwan. |
| **Xue 2022** | China |
| **Yang 2014** | China |
| **Yu 2020** | USA (3), Europe (3), Taiwan (2) |
| **Zhang 2023** | China |

**Supplementary Table S3: AMSTAR 2 Quality Assessment**

| **Review ID** | **1** | **2** | **3** | **4** | **5** | **6** | **7** | **8** | **9** | **10** | **11** | **12** | **13** | **14** | **15** | **16** | **Overall** |
| --- | --- | --- | --- | --- | --- | --- | --- | --- | --- | --- | --- | --- | --- | --- | --- | --- | --- |
| **Alvarez-Bueno 2020** | Yes | Yes | Yes | Yes | Yes | Yes | Yes | Yes | Yes | No | Yes | Yes | Yes | Yes | Yes | Yes | **High** |
| **Chiu 2021** | Yes | Yes | Yes | Partial yes | Yes | Yes | Yes | Yes | Yes | Yes | Yes | Yes | Yes | Yes | Yes | Yes | **High** |
| **Cooper 2015** | Yes | No | Yes | Partial yes | Yes | Yes | Partial yes | Yes | Yes | No | Yes | No | No | No | No | Yes | **Critically low** |
| **de Heus 2021** | Yes | Yes | Yes | Yes | Yes | Yes | Yes | Yes | Yes | No | Yes | Yes | Yes | Yes | Yes | Yes | **High** |
| **Gifford 2013** | Yes | Partial Yes | Yes | Partial Yes | Yes | No | No | Partial Yes | No | No | Yes | No | No | Yes | No | Yes | **Critically low** |
| **Guan 2011** | Yes | No | Yes | Partial Yes | No | Yes | No | Yes | Yes | No | No | No | No | Yes | No | Yes | **Critically low** |
| **He 2024** | Yes | Yes | Yes | Partial yes | Yes | Yes | No | Partial yes | Yes | No | Yes | Yes | Yes | No | No | Yes | **Critically low** |
| **Huang 2024** | Yes | Yes | Yes | Partial Yes | Yes | Yes | No | Yes | Yes | No | Yes | Yes | Yes | Yes | Yes | Yes | **Low** |
| **Iseli 2019** | Yes | Yes | No | partial yes | Yes | Yes | No | Yes | Yes | No | Yes | Yes | Yes | Yes | Yes | Yes | **Low** |
| **Jia 2021** | Yes | No | No | partial | Yes | Yes | No | Yes | Yes | No | Yes | Yes | Yes | Yes | No | Yes | **Critically low** |
| **Joyce 2024** | Yes | Yes | No | Partial Yes | Yes | Yes | No | Partial Yes | Partial Yes | No | Yes | No | No | No | No | Yes | **Critically low** |
| **Lee 2022** | Yes | Partial Yes | Yes | Partial Yes | No | No | No | Partial Yes | Partial Yes | No | Yes | Yes | Yes | Yes | No | No | **Critically low** |
| **Lennon 2019** | Yes | No | Yes | Partial Yes | Yes | Yes | No | Yes | Yes | No | Yes | Yes | Yes | Yes | Yes | No | **Critically low** |
| **Li 2016** | Yes | No | No | Partial Yes | No | No | No | Yes | Yes | No | Yes | Yes | Yes | Yes | Yes | Yes | **Critically low** |
| **Li 2019** | Yes | No | No | Partial Yes | Yes | Yes | No | Yes | Yes | No | Yes | Yes | Yes | Yes | Yes | Yes | **Critically low** |
| **Liang 2020** | Yes | Yes | Yes | Yes | Yes | Yes | No | Yes | Yes | No | Yes | Yes | Yes | Yes | Yes | Yes | **Low** |
| **Liu 2021** | Yes | No | No | Partial Yes | Yes | Yes | No | Yes | Yes | No | Yes | Yes | Yes | Yes | No | Yes | **Critically low** |
| **Meng 2014** | Yes | No | Yes | Partial Yes | No | No | No | Yes | Yes | No | Yes | Yes | Yes | Yes | Yes | Yes | **Critically low** |
| **Min 2020** | Yes | No | No | Partial Yes | Yes | Yes | No | Yes | Partial Yes | No | Yes | No | No | Yes | Yes | Yes | **Critically low** |
| **Ou 2020** | Yes | No | No | No | Yes | Yes | No | Yes | Yes | No | Yes | Yes | Yes | Yes | Yes | Yes | **Critically low** |
| **Pase 2012** | Yes | No | Yes | Partial Yes | Yes | Yes | No | Yes | No | No | Yes | No | No | Yes | No | No | **Critically low** |
| **Peters 2018** | Yes | Yes | Yes | Yes | Yes | Yes | No | Yes | Yes | No | Yes | Yes | No | Yes | Yes | Yes | **High** |
| **Power 2011** | Yes | No | No | Partial Yes | Yes | Yes | No | Yes | Yes | No | Yes | Yes | Yes | No | Yes | No | **Critically low** |
| **Sánchez-Nieto 2021** | Yes | No | No | Partial Yes | Yes | Yes | No | No | Yes | No | Yes | Yes | Yes | Yes | No | Yes | **Critically low** |
| **Sharp 2011** | Yes | No | Yes | Yes | Yes | Yes | No | No | No | No | Yes | No | No | No | No | Yes | **Critically low** |
| **Wang 2018** | Yes | Partial Yes | Yes | Partial Yes | No | No | Yes | No | Yes | No | Yes | Yes | No | Yes | No | Yes | **Critically low** |
| **Xu 2015** | Yes | No | Yes | Partial Yes | No | No | Yes | No | Partial Yes | No | Yes | Yes | No | Yes | Yes | No | **Critically low** |
| **Xue 2022** | Yes | No | No | Partial Yes | Yes | Yes | No | No | Yes | No | Yes | Yes | Yes | No | Yes | Yes | **Critically low** |
| **Yang 2014** | Yes | No | No | Partial Yes | Yes | Yes | No | No | Yes | No | Yes | Yes | No | No | No | Yes | **Critically low** |
| **Yu 2020** | Yes | Yes | Yes | Yes | Yes | Yes | Yes | No | Yes | Yes | Yes | Yes | Yes | Yes | Yes | Yes | **High** |
| **Zhang 2023** | Yes | No | No | Partial Yes | Yes | Yes | No | Partial Yes | Yes | No | Yes | No | No | Yes | No | No | **Critically low** |

# Supplementary Table S4: Calculation of Overlap of constituent studies

| **Variable** | **Times studies appeared in reviews** | **Number of rows** | **Number of reviews** | **Proportion**  **CCA = N – r/(r x c) - r** | **Percentage (%)** |
| --- | --- | --- | --- | --- | --- |
|  | **N** | **r** | **c** |  |  |
| **Hypertension/SBP/DBP and any outcome** | 288 | 209 | 20 | 0.019 | 2.0 |
| **Hypertension/SBP/DBP and dementia** | 171 | 99 | 12 | 0.066 | 6.6 |
| **Hypertension/SBP/DBP and cognitive decline/impairment** | 34 | 34 | 5 | 0 | 0 |
| **Hypertension/SBP/DBP and domains of cognition** | 53 | 49 | 4 | 0.027 | 2.7 |
| **BPV** | 55 | 33 | 3 | 0.3333 | 33.3 |
| **PWV** | 73 | 49 | 3 | 0.2449 | 24.5 |
| **OH** | 60 | 46 | 4 | 0.1014 | 10.1 |

Note: Lee et al. 2022^9^ not included because all included studies were all Mendelian Randomisation studies, which would not overlap with the other reviews.

**References**

1. Casiglia E and Jordan J. Orthostatic hypotension: new views for an old problem. *J Hypertens* 2017; 35: 947-949. 2017/03/30. DOI: 10.1097/hjh.0000000000001272.

2. Cooper C, Sommerlad A, Lyketsos CG, et al. Modifiable predictors of dementia in mild cognitive impairment: A systematic review and meta-analysis. *American Journal of Psychiatry* 2015; 172: 323-334. DOI: doi:<https://dx.doi.org/10.1176/appi.ajp.2014.14070878>.

3. Li JQ, Wang HF, Tan MS, et al. Risk factors for predicting progression from mild cognitive impairment to Alzheimer's disease: A systematic review and meta-analysis of cohort studies. *Journal of Neurology, Neurosurgery and Psychiatry* 2016; 87: 476-484. DOI: doi:<https://dx.doi.org/10.1136/jnnp-2014-310095>.

4. Liang JH, Lu L, Li JY, et al. Contributions of Modifiable Risk Factors to Dementia Incidence: A Bayesian Network Analysis. *Journal of the American Medical Directors Association* 2020; 21: 1592-1599.e1513. DOI: doi:<https://dx.doi.org/10.1016/j.jamda.2020.04.006>.

5. Yu JT, Xu W, Tan CC, et al. Evidence-based prevention of Alzheimer's disease: systematic review and meta-analysis of 243 observational prospective studies and 153 randomised controlled trials. *J Neurol Neurosurg Psychiatry* 2020; 91: 1201-1209. DOI: doi:10.1136/jnnp-2019-321913.

6. Wang ZT, Xu W, Wang HF, et al. Blood Pressure and the Risk of Dementia: A Dose-Response Meta-Analysis of Prospective Studies. *Curr Neurovasc Res* 2018; 15: 345-358. DOI: doi:10.2174/1567202616666181128114523.

7. Yang F, Liu QQ, Wang LJ, et al. Risk factors of vascular cognitive impairment among Chinese population: Meta-analysis. [Chinese]. *Journal of Jilin University Medicine Edition* 2014; 40: 626-632. DOI: doi:<https://dx.doi.org/10.13481/j.1671-587x.20140333>.

8. Guan JW, Huang CQ, Li YH, et al. No association between hypertension and risk for Alzheimer's disease: a meta-analysis of longitudinal studies. *J Alzheimers Dis* 2011; 27: 799-807. DOI: doi:10.3233/jad-2011-111160.

9. Lee L, Walker RM and Whiteley WN. Assessing the role of vascular risk factors in dementia: Mendelian randomization meta-analysis and comparison with observational estimates. *medRxiv* 2022; 25. DOI: doi:<https://dx.doi.org/10.1101/2022.02.23.22271334>.

10. Andrews SJ, Fulton-Howard B, O'Reilly P, et al. Causal Associations Between Modifiable Risk Factors and the Alzheimer's Phenome. *Ann Neurol* 2021; 89: 54-65. 2020/10/01. DOI: 10.1002/ana.25918.

11. Malik R, Georgakis MK, Neitzel J, et al. Midlife vascular risk factors and risk of incident dementia: Longitudinal cohort and Mendelian randomization analyses in the UK Biobank. *Alzheimer's & Dementia* 2021; 17: 1422-1431. DOI: <https://doi.org/10.1002/alz.12320>.

12. Lennon MJ, Makkar SR, Crawford JD, et al. Midlife Hypertension and Alzheimer's Disease: A Systematic Review and Meta-Analysis. *J Alzheimers Dis* 2019; 71: 307-316. DOI: doi:10.3233/jad-190474.

13. Li XY, Zhang M, Xu W, et al. Midlife Modifiable Risk Factors for Dementia: A Systematic Review and Meta-analysis of 34 Prospective Cohort Studies. *Curr Alzheimer Res* 2019; 16: 1254-1268. DOI: doi:10.2174/1567205017666200103111253.

14. Meng XF, Yu JT, Wang HF, et al. Midlife vascular risk factors and the risk of Alzheimer's disease: a systematic review and meta-analysis. *J Alzheimers Dis* 2014; 42: 1295-1310. DOI: doi:10.3233/jad-140954.

15. Ou YN, Tan CC, Shen XN, et al. Blood Pressure and Risks of Cognitive Impairment and Dementia: A Systematic Review and Meta-Analysis of 209 Prospective Studies. *Hypertension* 2020; 76: 217-225. DOI: doi:10.1161/hypertensionaha.120.14993.

16. Power MC, Weuve J, Gagne JJ, et al. The association between blood pressure and incident Alzheimer disease: a systematic review and meta-analysis. *Epidemiology* 2011; 22: 646-659. DOI: doi:10.1097/EDE.0b013e31822708b5.

17. Xu W, Tan L, Wang HF, et al. Meta-analysis of modifiable risk factors for Alzheimer's disease. *J Neurol Neurosurg Psychiatry* 2015; 86: 1299-1306. DOI: doi:10.1136/jnnp-2015-310548.

18. Sharp SI, Aarsland D, Day S, et al. Hypertension is a potential risk factor for vascular dementia: systematic review. *Int J Geriatr Psychiatry* 2011; 26: 661-669. DOI: doi:10.1002/gps.2572.

19. Gifford KA, Badaracco M, Liu D, et al. Blood pressure and cognition among older adults: a meta-analysis. *Arch Clin Neuropsychol* 2013; 28: 649-664. DOI: doi:10.1093/arclin/act046.

20. Sánchez-Nieto JM, Rivera-Sánchez UD and Mendoza-Núñez VM. Relationship between Arterial Hypertension with Cognitive Performance in Elderly. Systematic Review and Meta-Analysis. *Brain Sci* 2021; 11. DOI: doi:10.3390/brainsci11111445.

21. De Heus R and Tully P. The association of blood pressure variability with dementia and cognitive impairment: A systematic review and meta-analysis. *Journal of Hypertension* 2021; 39: e182. DOI: doi:<https://dx.doi.org/10.1097/01.hjh.0000746452.60467.27>.

22. Chiu TJ, Yeh JT, Huang CJ, et al. Blood pressure variability and cognitive dysfunction: A systematic review and meta-analysis of longitudinal cohort studies. *J Clin Hypertens (Greenwich)* 2021; 23: 1463-1482. DOI: doi:10.1111/jch.14310.

23. Jia P, Lee HWY, Chan JYC, et al. Long-Term Blood Pressure Variability Increases Risks of Dementia and Cognitive Decline: A Meta-Analysis of Longitudinal Studies. *Hypertension* 2021; 78: 996-1004. DOI: doi:10.1161/hypertensionaha.121.17788.

24. de Heus RAA, Tzourio C, Lee EJL, et al. Association Between Blood Pressure Variability With Dementia and Cognitive Impairment: A Systematic Review and Meta-Analysis. *Hypertension* 2021; 78: 1478-1489. DOI: doi:10.1161/hypertensionaha.121.17797.

25. Liu Q, Fang J, Cui C, et al. Association of Aortic Stiffness and Cognitive Decline: A Systematic Review and Meta-Analysis. *Front Aging Neurosci* 2021; 13: 680205. DOI: doi:10.3389/fnagi.2021.680205.

26. Pase MP, Herbert A, Grima NA, et al. Arterial stiffness as a cause of cognitive decline and dementia: a systematic review and meta-analysis. *Intern Med J* 2012; 42: 808-815. DOI: doi:10.1111/j.1445-5994.2011.02645.x.

27. Alvarez-Bueno C, Cunha PG, Martinez-Vizcaino V, et al. Arterial Stiffness and Cognition Among Adults: A Systematic Review and Meta-Analysis of Observational and Longitudinal Studies. *J Am Heart Assoc* 2020; 9: e014621. DOI: doi:10.1161/jaha.119.014621.

28. Min M, Shi T, Sun C, et al. The association between orthostatic hypotension and cognition and stroke: a meta-analysis of prospective cohort studies. *Blood Pressure* 2020; 29: 3-12. DOI: doi:<https://dx.doi.org/10.1080/08037051.2019.1689808>.

29. Peters R, Anstey KJ, Booth A, et al. Orthostatic hypotension and symptomatic subclinical orthostatic hypotension increase risk of cognitive impairment: an integrated evidence review and analysis of a large older adult hypertensive cohort. *Eur Heart J* 2018; 39: 3135-3143. DOI: doi:10.1093/eurheartj/ehy418.

30. Iseli R, Nguyen VTV, Sharmin S, et al. Orthostatic hypotension and cognition in older adults: A systematic review and meta-analysis. *Exp Gerontol* 2019; 120: 40-49. DOI: doi:10.1016/j.exger.2019.02.017.
